# The quality of skilled birth attendants in Nepal: High aspirations and ground realities

**DOI:** 10.1371/journal.pone.0214577

**Published:** 2019-04-04

**Authors:** Ruma Rajbhandari, Shovana Rai, Sejal Hathi, Rita Thapa, Indra Rai, Anil Shrestha

**Affiliations:** 1 Division of Global Health Equity, Brigham and Women’s Hospital, Harvard Medical School, Boston, Massachusetts, United States of America; 2 Nick Simons Institute, Kathmandu, Nepal; 3 Stanford Medical School, Palo Alto, California, United States of America; Golden Community, NEPAL

## Abstract

**Background:**

While Nepal’s maternal mortality ratio (MMR) has improved overall, the proportion of maternal deaths occurring in health facilities and attended to by skilled birth attendants (SBAs), has nearly doubled over 12 years. Although there are numerous socioeconomic, environmental and other factors at play, one possible explanation for this discrepancy between utilization of skilled maternal care services and birth outcomes lies in the quality of care being provided by SBAs. The objective of this study is to determine how competent SBAs are after training, across multiple settings and facility types in Nepal.

**Methods:**

We used a quantitative cross-sectional analysis to evaluate a sample of 511 SBAs, all female, from 276 sub-health posts (SHP), health posts (HP), primary healthcare centers (PHC), and district and regional hospitals in the mountain, hill, and terai districts of Nepal. Any SBA actively employed by one of these health facilities was included. SBAs who had received less than three months of training were excluded. Outcomes were measured using SBAs’ scores on a standardized knowledge assessment, clinical skills assessment, and monthly delivery volume, particularly as it compared with the WHO’s recommendation for minimum monthly volume to maintain competence.

**Results:**

SBAs on average exhibit a deficiency of both knowledge and clinical skills, failing to meet even the 80-percent standard that is required to pass training (knowledge: 75%, standard deviation 12%; clinical skills: 48%, standard deviation 15%). Moreover, SBAs are conducting very few deliveries, with only 7 percent (38/511) meeting the minimal volume recommended to maintain competence by the WHO, and a substantial fraction (70/511, 14%) performing an average of no monthly deliveries at all.

**Conclusions:**

Taken together, our findings suggest that while countries like Nepal have made important investments in SBA programs, these healthcare workers are failing to receive either effective training or sufficient practice to stay clinically competent and knowledgeable in the field. This could in part explain why institutional deliveries have generally failed to deliver better outcomes for pregnant women and their babies.

## Introduction

By 2015, the global maternal mortality ratio (MMR) had declined to 216 deaths per 100,000 live births, a roughly 44 percent decrease since 1990 [1, 2]. This decline fell far short of the three-quarters reduction targeted by the Millennium Development Goals (MDGs). It also belied gross disparities between urban and rural, developed and developing regions—some with MMRs that in fact paradoxically increased over 25 years [1, 3, 4]. Interestingly, while the MMR target was missed, women’s access to, and usage of, maternal care services soared, with more than 70 percent of births delivered by skilled birth attendants (SBAs) in 2015 [2, 3]. The discrepancy between access to care and health outcomes is likely explained by multiple systemic, economic, social, and physical factors that vary from one community to another, but may also suggest a critical gap in quality of care provided by SBAs. Merely training more SBAs will not suffice: more attention needs to be directed at their knowledge and skills and how that translates to the care that they provide for women and infants.

Several studies have attempted to measure the competence of SBAs. The first systematic assessment, conducted from 2001 to 2002 across four countries—Benin, Ecuador, Jamaica, and Rwanda—uncovered “a wide gap” among SBAs “between current evidence-based standards and provider competence [5].” Subsequent studies have echoed these results, documenting severe deficiencies of both clinical skills and knowledge of SBAs in Nepal, Bangladesh, Afghanistan, Pakistan, and Indonesia [6–10]. As countries continue to invest in SBA training—targets for which have only heightened under the Sustainable Development Goals—the efficacy of that training and the extent to which SBA skills are retained in the field become ever more important questions.

Nepal offers a prime case study in this challenge. On the one hand, both the MMR and the proportion of births attended by SBAs have achieved substantial gains over the last 25 years. Indeed, Nepal transcended lackluster performance globally to become one of the first countries to meet its maternal mortality targets in 2013, rapidly being celebrated as a redemptive success story [11]. The government has proven a consistent partner in this progress, having enacted a National Policy on Skilled Birth Attendants that has overseen the training of 7,000 SBAs since 2006 [12–16]. A more critical subnational analysis reveals persistent inequalities: Maternal deaths remain disproportionately high in the mountainous district, compared with the hills and lowland Terai, as well as among lower socioeconomic classes. Moreover, the proportion of these deaths occurring in health facilities, where SBAs work, as opposed to at home or in transit has nearly doubled over 12 years, suggesting that institutional delivery is not synonymous with quality [12, 13, 17]. These findings cast a focus on SBAs’ competence in the field, as well as those enabling factors required for them to maintain their skills in basic and emergency obstetric care. A 2008/2009 Nepal maternal mortality study found that, while maternal deaths at home decreased from 67% in 1998 to 40% in 2008, maternal deaths in facilities increased from 21% to 41% during the same time period [17]. It is important to note that environmental, socioeconomic, and other factors likely play a role in the discrepancy between increased facility births and maternal mortality, including that more women may go to a facility when they have complications but too late for SBA intervention to be effective [17].

The Nepal SBA training program is a 3-month in-service training program for nurses, doctors, and midwives, based on a checklist of 27 core skills and abilities for safe birth, which includes management of normal deliveries as well as potential complications. The objective of this study is to measure the level of knowledge, the degree of skill retention, and the productivity of a sample of Nepal’s SBAs. We were particularly interested in post-training follow up in the field: what do SBAs actually do after SBA training? We thus posed three questions: 1) what level of knowledge do SBAs exhibit, and which demographic and environmental factors correlate with higher knowledge scores? 2) What level of clinical skills do SBAs exhibit, and which factors correlate with higher skills? And, given the proven association between case volume and patient outcomes, 3) how many deliveries are SBAs performing per month, and how many of them are meeting World Health Organization (WHO) guidelines for minimum volume to maintain competence [18–21]? What are predictors of higher delivery volume among SBAs?

## Methods

We used a quantitative retrospective cross-sectional analysis to evaluate a sample of 511 SBAs in Nepal. The Nick Simons Institute (NSI), via its Follow-up Enhancement Program (FEP), and the Government of Nepal’s National Health Training Center (NHTC) collected data from 2013 to 2016, attempting to follow up with 30% of SBA trainees, per NHTC recommendation. Districts were purposively sampled to represent the three ecological zones: hill, mountain, terai. We randomly chose districts from each eco-zone. The unit of randomization was the district, but individual SBA counts were used for analyses. We chose 3 districts in the Mountain region, 5 from the Hills and 7 from the Terai (S2 Table). Higher numbers of districts were chosen in the Terai versus the Mountains because a larger proportion of the Nepalese populations lives in the Terai. We then visited all of the health facilities (hospitals, PHCs, HPs, SHPs) in a given district that had skilled birth attendants working at them and carried out assessments on every one of the SBAs. We excluded SBAs who had received less than three months of training to ensure only fully trained SBAs were included, and only female SBAs are included because there were no male SBA trainees in the program. 511 SBAs remained after all inclusion and exclusion criteria were applied. We obtained ethical approval from the Nepal Health Research Council of Nepal (reference number 2410).

### Ethics statement

Please note that this was a retrospective observational study of data already collected by NSI in the context of the FEP. Data were not collected for the sole purpose of research but rather as part of the FEP program carried out in conjunction with the National Health Training Center of Nepal. Approvals for the FEP program were obtained from the National Health Training Center, Department of Health Services. Since the FEP program was carried out in collaboration with the National Health Training Center, data collection was a routine part of the government program and individual consent from SBAs was verbally obtained.

### Data collection

The paper FEP tool was administered to individual SBAs by SBA trainers, who are doctors and nurses who have completed an SBA course, Clinical Training Skills course, and National Health Training Center certification, and have 1–4 years of SBA training experience, with the assistance of NSI and public health nurses. The SBA FEP tool was drafted by SBA trainers, Support for Safer Motherhood Project, and Family Health Division and was pretested in five districts, the findings of which were disseminated to stakeholders and used to revised the tool. The assessment, which included background information, knowledge and clinical assessment, clinical decision making, infection prevention, and an enabling environment checklist, took approximately 5 hours for each SBA to complete. SBA trainers also conducted guided qualitative interviews with the participants, but this information was not robust enough to analyze for the purposes of this study. SBA trainers were not blinded, as the FEP was not performed only for research purposes.

We collected descriptive information including demographic characteristics like SBAs’ age, position (auxiliary nurse midwife versus staff nurse), recruitment status (permanent position versus temporary hire); level of experience (the number of months of both total nursing and SBA experience); training site type (whether continuously monitored and supported by the Nick Simons Institute, or not; support entailed financial and technical support with frequent monitoring visits to ensure a high level of quality of care and teaching at the sites); location (mountain, hill or terai district) and facility (hospital versus lower level facility like PHC, HP and SHP); and environmental characteristics: the presence or absence at the SBA’s facility of each of a comprehensive set of 74 structural and inventory items that enable basic emergency obstetric care. We assessed level of knowledge of procedures and managing complications using a 20-question multiple-choice survey. We assessed level of clinical skills using standardized checklists on anatomical models and cases for use of the partograph, normal and vacuum delivery, newborn resuscitation, referral, pregnancy complication management. Normal delivery, vacuum delivery, and newborn resuscitation were assessed via demonstration on a mannequin; and partograph, postpartum hemorrhage management skills, eclampsia, and shock management were assessed via case-based discussions. We developed both the knowledge survey and clinical skills checklists using a committee of experts and WHO and JHPIEGO guidelines. The enabling environment was evaluated via FEP trainer assessment of a comprehensive checklist including infrastructure, equipment, and drugs at each facility. We assessed the total monthly deliveries for each SBA by reviewing each facility’s delivery logs, and collected data on number of normal deliveries, vacuum deliveries, breech deliveries, and the management of any of the following 7 complications over a 3-month period: postpartum hemorrhage, antepartum hemorrhage, eclampsia, episiotomy, manual removal of the placenta, post-abortion care, and newborn resuscitation.

### Data analysis

Data collected from the FEP tool from 2013 to 2016 was later reviewed by NSI and NHTC staff for analysis, as this data was initially collected with the intention of quality improvement and coaching or enhancement of SBAs, rather than research. While both qualitative and quantitative data were collected as part of the FEP, only quantitative data were analyzed for the purpose of this study. We recorded all quantitative data in Excel and analyzed it with Stata Version SE14. Our main outcomes were SBAs’ 1) level of knowledge, 2) level of clinical skills and 3) total number of monthly deliveries. For level of knowledge, we calculated a mean percentage score, range, and standard deviation based on the number of questions answered correctly. For level of clinical skills, we calculated mean percentage scores with range and standard deviation for each of the eight domains tested, based on the number of checklist items within each domain that were successfully demonstrated. For monthly deliveries, we calculated the percentage of SBAs in each of four categories: 0, up to 4, between 4 and 15, and at least 15 monthly deliveries. We tabulated similar statistics for delivery complications. For the enabling environment, we conducted a literature review to identify which of the 74 indicators studied were deemed most essential in similar settings. We selected a final list of 17 indicators and calculated a mean percentage score according to the fraction of these present at any SBA’s health facility. This list spanned three categories: 1) general requirements—electricity, water, and a toilet inside the labor room; 2) routine delivery care—a partograph, fetoscope, baby weighing machine, blood pressure instrument, soap, gloves, an autoclave, and a standard delivery set; and 3) basic emergency obstetric and newborn care—oxytocin, magnesium sulfate, a vacuum delivery set, tear repair set, newborn resuscitation set, and I.V. set.

We used the t-test to compare continuous variables, and the chi-square test to conduct between-group comparisons for categorical variables. We performed univariable and multivariable linear regressions to identify independent factors associated with 1) knowledge scores, 2) clinical skills scores, and 3) volume of monthly deliveries. To compare results between hospitals and lower-level facilities, we performed a sub-group analysis of the delivery volume outcome by facility type.

### Patient involvement and dissemination

The focus of this study was the healthcare worker, specifically the skilled birth attendant. Thus, we did not involve patients in the development of the research question or in the design or writing up of the study. We will make the study’s findings publicly available on the Nick Simons Institute’s website. Additionally, we will disseminate the results of the study to the relevant governmental bodies in Nepal, all in coordination with the National Health Training Center.

## Results

### Demographic characteristics of SBAs

511 SBAs were sampled from 276 health institutions, across 15 districts, in all three of Nepal’s ecological zones (S1 Table). Of the total 511 SBAs, 75% of them worked in lower-level facilities (PHC, HP, or SHP) and 25% worked in hospitals. More than half (57%) were between the ages of 20 and 40 years, with the mean age of 33 years ranging 19–59 years (Table 1).

**Table 1:** Descriptive statistics of skilled birth attendants (SBAs) by facility type, n = 511.

Variables	Count (%) of SBAs		
	Lower-level facilities*	Hospitals	Aggregate
	n = 383 (75%)	n = 128 (25%)	n = 511 (100%)
**Mean age [SD]**	33 [9]	33 [9]	33 [9]
**Age categories: <29**	161 (42%)	53 (41%)	214 (42%)
**30–39**	130 (34%)	51 (40%)	181 (35%)
**40–49**	73 (19%)	12 (9%)	85 (17%)
**> = 50**	19 (5%)	12 (9%)	31 (6%)
**Position:**			
**Staff Nurse**	9 (2%)	37 (29%)	46 (9%)
**Auxiliary Nurse Midwife**	374 (98%)	91 (71%)	465 (91%)
**Recruitment:**			
**Permanent**	185 (48%)	76 (59%)	261 (51%)
**Temporary**	197 (52%)	52 (41%)	249 (49%)
**Total Nursing Experience, months [SD]**	115 [89]	124 [105]	117 [93]
**Total SBA Experience, months [SD]**	34 [25]	36 [31]	35 [26]
**Training Site:**			
**Continuously monitored and supervised sites**	56 (15%)	27 (21%)	83 (16%)
**Other sites with lower levels of monitoring and supervision**	327 (85%)	101 (79%)	428 (84%)
**District:**			
**Mountains**	54 (14%)	15 (12%)	69 (14%)
**Hills**	127 (33%)	31 (24%)	158 (31%)
**Terai**	202 (53%)	82 (64%)	284 (56%)

Values presented as Count (%) of SBAs; Mean [SD]

* Lower-level facilities include PHC, HP, and SHP

91% of SBAs were auxiliary nurse midwives (ANMs), and 9% were staff nurses. Staff nurses were approximately 15 times more represented in hospitals than in lower-level facilities (29% vs 2%).

SBAs were split nearly evenly between permanent and temporary nurses overall—permanent nurses made up 51% of SBAs and temporary nurses made up 49% of SBAs. However, hospitals had a higher percentage of permanent SBAs (59%) compared to temporary ones (41%) while the opposite was true for lower level facilities (permanent 48%, temporary 52%).

On average SBAs had accumulated nearly ten years of nursing experience, three of which comprised SBA experience. At both hospitals and lower-level facilities, less than a fifth of SBAs had been trained at a continuously monitored and supported training site, though hospital-based SBAs were 6% more likely than those at lower-level facilities. At the time of study, 56% of SBAs were working at facilities in the Terai, 31% in the Hills, and 14% in the Mountains.

### Enabling environment indicators

An examination of each SBA’s work environment revealed a high mean score on the index of enabling environment indicators determined to be most essential to quality maternal care. Specifically, SBAs had access to on average 83% [SD 10] (Table 2) of the 17 key enabling environment indicators, with at least 99% of them in settings with a fetoscope, baby weighing machine, gloves, and oxytocin in stock. By contrast, only 23% of SBAs could point to a toilet in the labor room, while only 27% could obtain a standard delivery set on-site. Surprisingly, only 78% of SBAs worked in a setting with readily available drinking water. Between facilities, hospitals achieved total environmental index scores on average eight percent higher (89% [SD 7]) than their lower-level counterparts (81% [SD 10]). The greatest differences emerged for availability of basic infrastructural components like electricity (91% at lower-level facilities versus 100% at hospitals, water (75% versus 88%), and a toilet in the labor room (22% vs 27%) as well as the requirement for routine and basic emergency obstetric care: partograph (83% at lower level facilities compared to 98% at hospitals), standard delivery set (24% vs 38%) and vacuum delivery set (35% vs 94%).

**Table 2:** Key enabling environment index scores (%) by facility type.

	Lower-level facilities §	Hospitals	Aggregate
	n = 364	n = 128	n = 492
**Total Score, % [SD]**	81 [10]	89 [7]	83 [10]
**General Requirements**			
**Electricity**	331 (91%)	128 (100%)	458 (93%)
**Water**	273 (75%)	113 (88%)	384 (78%)
**Toilet**	80 (22%)	35 (27%)	113 (23%)
**Routine Delivery Care**			
**Partograph**	302 (83%)	125 (98%)	428 (87%)
**Fetoscope**	364 (100%)	128 (100%)	492 (100%)
**Baby weighing machine**	360 (99%)	128 (100%)	487 (99%)
**Blood pressure instrument**	360 (99%)	120 (94%)	477 (97%)
**Soap**	357 (98%)	122 (95%)	477 (97%)
**Gloves**	364 (99%)	128 (100%)	487 (99%)
**Autoclave**	328 (90%)	127 (99%)	453 (92%)
**Standard delivery set**	87 (24%)	49 (38%)	133 (27%)
Basic EmONC*			
**Oxytocin**	360 (99%)	128 (100%)	487 (99%)
**Vacuum delivery set**	124 (35%)	120 (94%)	246 (50%)
**Tear repair set**	288 (79%)	115 (90%)	403 (82%)
MgSO4*	331 (91%)	128 (100%)	458 (93%)
**Newborn resuscitation set**	328 (90%)	128 (100%)	458 (93%)
**I.V. set**	349 (96%)	119 (93%)	467 (95%)

Values presented as Count (%) of SBAs; Mean [SD]

*EmONC: Emergency obstetric and newborn care, MgSO4: Magnesium sulfate

^§^Please note that the “n” is smaller here due to missing data regarding environmental factors for some lower level health facilities.

### Knowledge of SBAs and predictors of knowledge

SBAs achieved a total mean score of 75% [SD 12] on the written knowledge assessment section of the FEP data collection tool, with roughly comparable performance between hospitals and lower-level facilities (Table 3). On multivariable analysis, SBAs’ age, position and access to key enabling environment indicators proved predictive of their knowledge scores (Table 4). SBAs above the age of 50 received knowledge scores that were on average 8.88 percentage points lower than those under 30 years (95% CI -15.48 to -2.28, p = 0.009). Auxiliary nurse midwives received scores that were on average 4.72 percentage points lower than staff nurses (95% CI -8.59 to -0.86, p = 0.017). On average, a one-point increase in the enabling environment index for an SBA’s health facility correlated with a 1.48 percentage point increase in knowledge score for that SBA (95% CI 0.80 to 2.16, p<0.0001).

**Table 3:** Knowledge and clinical skills mean scores (%) of SBAs, n = 511.

	Lower-level facilities	Hospitals	Aggregate
**Knowledge Score, total**	74 [12]	78 [11]	75 [12]
**Clinical Skills Score, total**	46 [15]	51 [16]	48 [1]
**Partograph**	40 [27]	48 [28]	42 [28]
**Normal delivery**	55 [19]	56 [22]	55 [19]
**Vacuum delivery**	22 [27]	39 [29]	26 [28]
**Newborn resuscitation**	51 [25]	53 [25]	52 [25]
**Eclampsia management**	50 [24]	54 [24]	51 [24]
**Referral**	36 [24]	32 [23]	35 [24]
**Shock management**	33 [20]	40 [22]	35 [21]
**Post-partum hemorrhage management**	83 [17]	87 [15]	84 [17]

Values presented as Mean [SD]

**Table 4:** Multivariate regression analysis of predictors of knowledge scores.

	Knowledge Score (%)	
	(Adjusted)	
	β (95% CI)	p value
**Age Range**		
**0–29**	ref	
**30–39**	-1·63 (-4·19 to 0·93)	0·212
**40–49**	-3·53 (-7·68 to 0·62)	0·095
**50+**	-8·88 (-15·48 to -2·28)	0·009
**Position (ANM)**	-4·72 (-8·59 to -0·86)	0·017
**Recruitment (temporary)**	-2·03 (-4·49 to 0·44)	0·107
**Total Months of Nursing Experience**	-0·01 (-0·03 to 0·01)	0·299
**Total Months of SBA Experience**	0·01 (-0·04 to 0·05)	0·753
**Total Number of Deliveries**	0·03 (-0·06 to 0·12)	0·5
**Training at continuously monitored and supervised site**	2·22 (-0·56 to 5·00)	0·118
**Presence of Key Enabling Indicators**	1·48 (0·80 to 2·16)	<0·0001
**Facility Type (hospital)**	0·64 (-2·06 to 3·33)	0·644
**District:**		
**Mountains**	ref	
**Hills**	-1·04 (-4·42 to 2·33)	0·545
**Terai**	-0·84 (-3·99 to 2·32)	0·603

### Clinical skills of SBAs and predictors of clinical skills

Considered both independently and relative to their knowledge assessments, SBAs performed poorly on their clinical skills, with a total mean score of 48% [SD 15] across facilities (Table 3). SBAs at hospitals did slightly better than their counterparts at lower-level facilities, achieving a mean score of 51% [SD 16] versus 46% [SD 15]. Among the clinical skills tested, SBAs demonstrated the greatest aptitude in managing post-partum hemorrhages with a mean score of 84% [SD 17], while they particularly struggled in performing vacuum deliveries with a mean score of 26% [SD 28]). This was especially true at lower-level health facilities where the average vacuum delivery scores was 22% [SD 27] compared with hospitals’ mean score of 39% [SD 29]. Similarly, management of shock was poor overall, and worse for SBAs working at lower-level health facilities with a mean score of 33% [SD 20] than for those at hospitals with a mean of 40% [SD 22]. The only clinical skill at which SBAs demonstrated superiority at lower level facilities was referral management, with an average score of 36% [SD 24] at lower-level facilities versus 32% [SD 23%] at hospitals.

On multivariable analysis, SBAs’ age, position, training site, and access to enabling environment indicators proved predictive of their clinical skills scores (Table 5). SBAs above the age of 50 received clinical skills scores that were on average 11.64 percentage points lower than those under 30 years (95% CI -19.76 to -3.52, p = 0.005). Auxiliary nurse midwives posted clinical skills scores that were 8.56 percentage points lower than staff nurses (95% CI -13.32 to -3.81, p<0.0001). SBAs trained at continuously monitored and supervised training sites achieved average clinical skills scores 4.85 percentage points higher than those trained at other sites with lower levels of monitoring and supervision (95% CI 1.43 to 8.28, p = 0.006). On average, a one-point increase in the enabling environment index for an SBA’s health facility correlated with a 2.31-percentage point increase in clinical skills scores for that SBA (95% CI 1.47 to 3.14, p<0.0001).

**Table 5:** Multivariate regression analysis of predictors of clinical skills scores.

	Clinical Skills Score (%)	
	(Adjusted)	
	β(95% CI)	p value
**Age Range**		
**0–29**	ref	
**30–39**	0·26 (-2·90 to 3·41)	0·873
**40–49**	-2·18 (-7·28 to 2·92)	0·402
**50+**	-11·64 (-19·76 to -3·52)	0·005
**Position (ANM)**	-8·56 (-13·32 to -3·81)	<0·0001
**Recruitment (temporary)**	-2·23 (-5·26 to 0·80)	0·149
**Total Months of Nursing Experience**	0·01 (-0·01 to 0·03)	0·387
**Total Months of SBA Experience**	-0·03 (-0·09 to 0·02)	0·202
**Total Number of Deliveries**	-0·01 (-0·12 to 0·10)	0·872
**Training at continuously monitored and supervised site**	4·85 (1·43 to 8·28)	0·006
**Presence of Key Enabling Indicators**	2·31 (1·47 to 3·14)	<0·0001
**Facility Type (hospital)**	-2·26 (-5·58 to 1·06)	0·181
**District:**		
**Mountains**	ref	
**Hills**	-1·66 (-5·81 to 2·49)	0·432
**Terai**	1·61 (-2·28 to 5·49)	0·417

### Deliveries and predictors of deliveries

**Fig 1:**
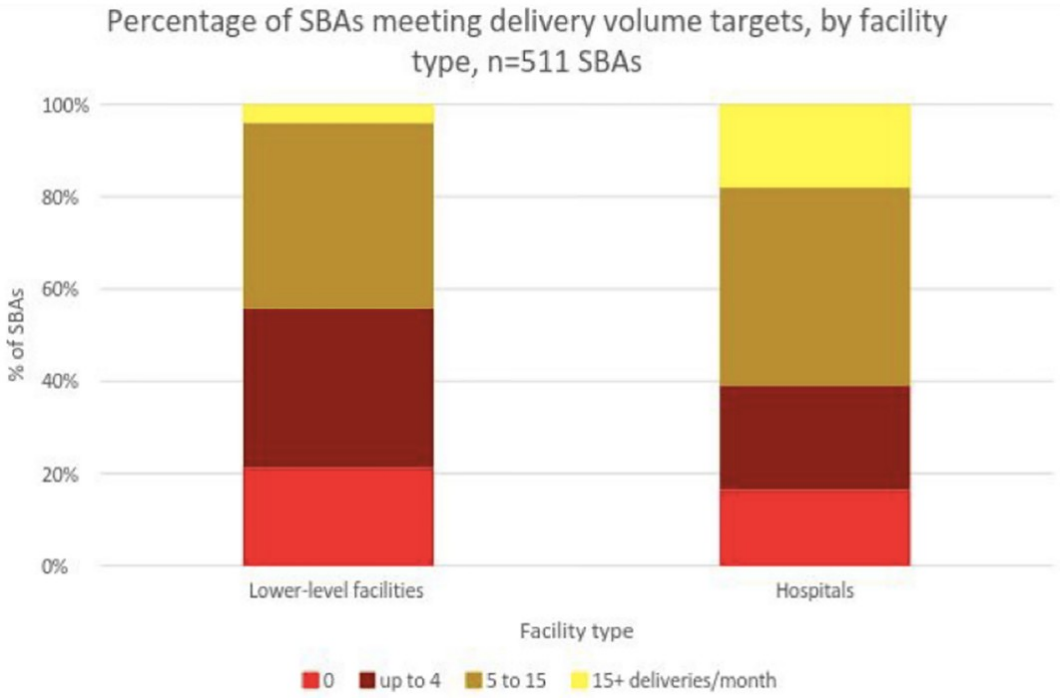
Percentage of SBAs meeting delivery volume targets by facility type, n = 511.

Each month, 1108 deliveries occurred in hospitals, 196 in PHCs, 957 in HPs, and 25 in sub-health posts across all three ecological zones (S2 Table). An analysis of monthly delivery volume revealed that only 9% of SBAs on average were meeting the WHO’s guideline of 15 deliveries per month to maintain competence. This number was significantly lower for SBAs working at lower-level facilities than for those at hospitals: 4% versus 18%, respectively (Fig 1). By contrast, a substantial fraction of SBAs—21% at lower-level facilities and 16% at hospitals—conducted zero monthly deliveries. Approximately 42% of SBAs performed between one and four monthly deliveries on average.

Of the SBAs who performed an average of zero deliveries per month, mean age was slightly higher than for the population at large, standing at 37 years [9], compared with 33 years for SBAs in aggregate (Table 6). Average total nursing and total SBA experience were accordingly greater as well, at 160 months [105] and 41 months [29]. 91% of this subset of SBAs were ANMs, and 85% were trained at sites with lower levels of monitoring and supervision, similar to the aggregate sample. Slightly more than in aggregate (59% vs 53%) practiced in the Terai district. The most significant difference lay in recruitment: compared with temporary contract nurses, permanent nurses comprised a disproportionately higher percentage of SBAs conducting zero monthly deliveries than they did of SBAs in aggregate (64% vs 51%, respectively).

**Table 6:** Descriptive statistics of SBAs that performed 0 monthly deliveries, n = 70.

Variables	Count (%) of SBAs
	n = 70
**Age mean [SD]**	37 [9]
**Age categories:**	
**<29**	15 (21%)
**30–39**	27 (39%)
**40–49**	19 (27%)
**> = 50**	9 (13%)
**Position:**	
**Staff Nurse**	6 (9%)
**Auxiliary Nurse Midwife**	64 (91%)
**Recruitment:**	
**Permanent**	45 (64%)
**Temporary**	25 (36%)
**Total Nursing Experience, months [SD]**	160 [105]
**Total SBA Experience, months [SD]**	41 [29]
**Training Site:**	
**Continuously monitored and supervised sites**	11 (16%)
**Other sites with lower levels of monitoring and supervision**	59 (84%)
**Facility Type:**	
**Hospital**	18 (26%)
**Lower-level Facility**	52 (74%)
**District:**	
**Mountains**	11 (16%)
**Hills**	18 (26%)
**Terai**	41 (59%)
**Total Knowledge Score (%, [SD])**	73 [11]
**Total Clinical Skills Score (%, [SD])**	70 [46]
**Enabling Environment Index Score (%, [SD])**	83 [11]

Values presented as Count (%) of SBAs; Mean [SD]

**Fig 2:**
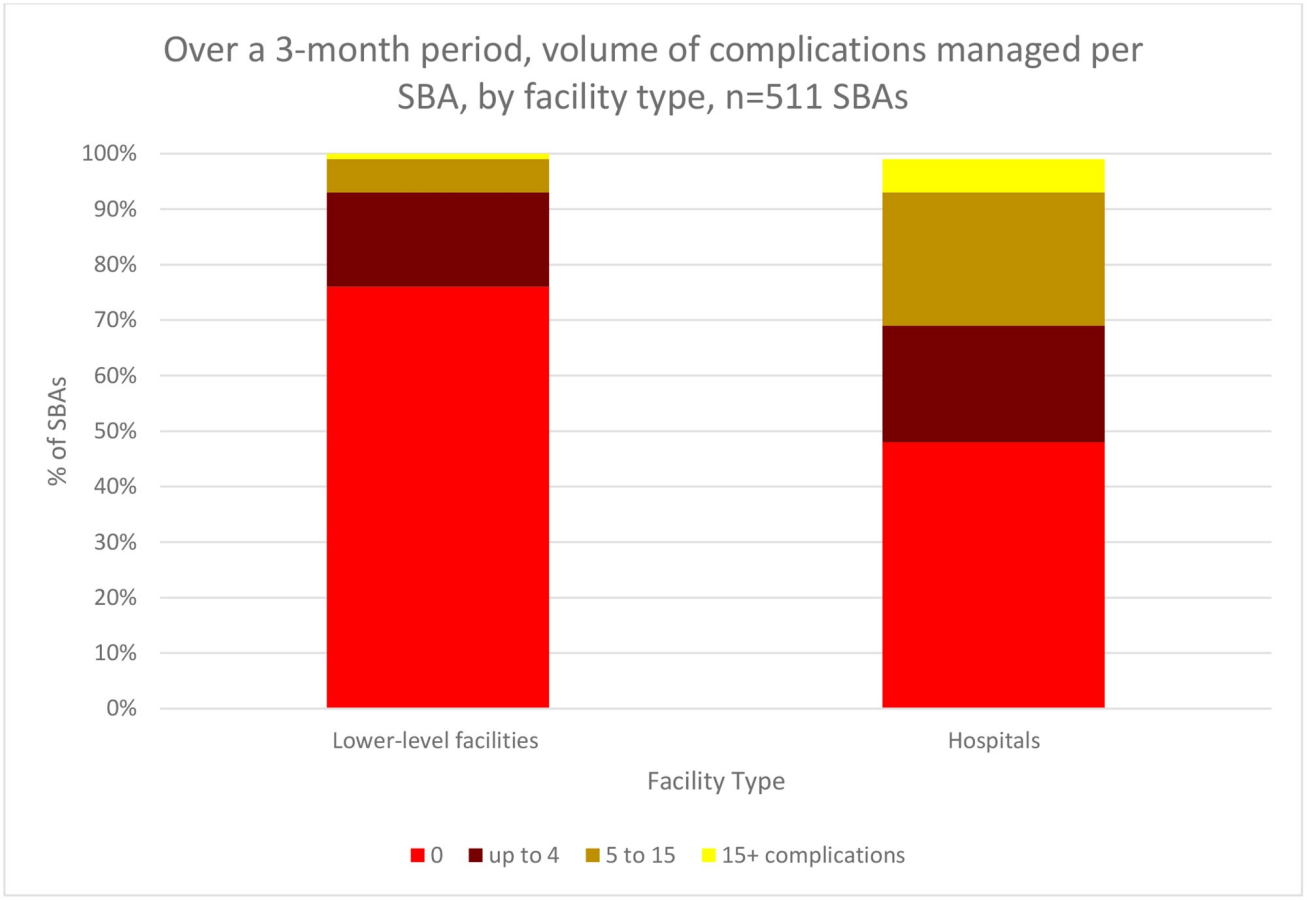
Over a 3-month period, volume of complications managed per SBA, by facility type, n = 511 SBAs.

Over a three-month period, a total of 821 complications were managed in our sample (S3 Table). The majority of SBAs managed zero complications (Fig 2), with this proportion higher for those based at lower-level facilities (76%) than at hospitals (48%). By contrast, only 1 percent of SBAs at lower-level facilities and 6 percent of those at hospitals managed at least 15 complications over the three months. The most frequently managed complications were episiotomies and newborn resuscitations; the least frequently managed: antepartum hemorrhage and post-abortion care.

On multivariable analysis, statistically significant predictors of SBA monthly delivery volume were training at a continuously monitored and supervised site and the type of facility and type of district in which SBAs practiced (Table 7). Of note, this multivariable analysis included the additional control variable of expected number of deliveries at the SBA’s health facility, which was calculated according to the demographic characteristics of each catchment population. On average, SBAs who were trained at a continuously monitored and supervised site performed an additional 6 deliveries per month over those trained at other sites with lower levels of monitoring and supervision [5.99 (3.06 to 8.92), p<0.001]. SBAs working at hospitals performed an additional 5 deliveries per month over those at lower-level facilities [5.06 (2.18 to 7.95), p = 0.001]. SBAs working in the Terai district performed 5 deliveries per month more on average than their counterparts in the mountain district [5.19 (1.95 to 8.44), p = 0.002].

**Table 7:** Multivariate regression analysis of predictors of total monthly deliveries.

	Total deliveries	
	(Adjusted)	
	β(95% CI)	p value
**Age Range**		
**0–29**	ref	
**30–39**	0·56 (-2·15 to 3·27)	0·684
**40–49**	3·29 (-1·15 to 7·73)	0·146
**50+**	-2·69 (-9·69 to 4·30)	0·449
**Position (ANM)**	1·39 (-2·80 to 5·59)	0·515
**Recruitment (temporary)**	1·40 (-1·23 to 4·03)	0·296
**Total Months of Nursing Experience**	-·005 (-0·03 to 0·02)	0·664
**Total months of SBA Experience**	0·03 (-0·02 to 0·07)	0·256
**Total Knowledge Score (%)**	0·04 (-0·06 to 0·15)	0·392
**Total Clinical Skills Score (%)**	-0·02 (-0·10 to 0·06)	0·636
**Training at continuously monitored and supervised site**	5·99 (3·06 to 8·92)	<0·0001
**Presence of Key Enabling Indicators**	0·41 (-0·34 to 1·16)	0·281
**Facility Type (hospital)**	5·06 (2·18 to 7·95)	0·001
**District:**		
**Mountains**	ref	
**Hills**	1·60 (-1·92 to 5·12)	0·372
**Terai**	5·19 (1·95 to 8·44)	0·002
**Expected Deliveries per SBA**	0·03 (-0·01 to 0·08)	0·124

On subgroup analysis by facility type, there were significant differences between hospitals and lower-level facilities in those factors that proved predictive of monthly delivery volume (Table 8). For lower-level health facilities, an SBA’s recruitment status, access to enabling environment indicators, and geographical district of practice were positively predictive of monthly delivery volume; age over 50 years was negatively predictive. For example, at lower level health facilities, temporary SBAs conducted 1.95 more deliveries than permanent SBAs (p = 0.003); SBAs over the age of 50 conducted 3.6 less deliveries than SBAs less than 30 years old (p = 0.026); a one point increase in the enabling environment index meant a 0.46 increase in number of deliveries (p = 0.007); and SBAs based in the Terai conducted 2.90 more deliveries than those based in the mountains (p<0.0001). For hospitals, by contrast, only an SBA’s geographical district of practice and training at a continuously monitored and supervised site were positively predictive; no factors were negatively predictive. For example, at hospitals, SBAs who were trained at a continuously monitored and supervised site carried out almost 18 more deliveries than those trained at other sites with lower levels of monitoring and supervision (p<0.0001) and SBAs based in the Terai carried out almost 12 more deliveries than those based in the mountains (p = 0.052).

**Table 8:** Multivariate regression analysis of predictors of total delivery, by facility type.

	Lower-level facilities		Hospitals	
	β(95% CI)	p value	β(95% CI)	p value
**Age Range**				
**0–29**	ref		ref	
**30–39**	-0.57 (-1.83 to 0.68)	0.367	3.94 (-6.76 to 14.63)	0.467
**40–49**	-1.39 (-3.39 to 0.61)	0.173	19.14 (-0.71 to 39.00)	0.059
**50+**	-3.60 (-6.76 to -0.43)	0.026	12.01 (-20.28 to 44.30)	0.463
**Position (ANM)**	3.12 (-0.49 to 6.73)	0.09	-0.05 (-9.02 to 8.92)	0.991
**Recruitment (temporary)**	1.95 (0.69 to 3.22)	0.003	1.04 (-7.80 to 9.88)	0.816
**Total Months of Nursing Experience**	0.002 (-0.01 to 0.01)	0.609	-0.04 (-0.14 to 0.05)	0.372
**Total months of SBA Experience**	0.02 (-0.01 to 0.04)	0.125	0.07 (-0.07 to 0.21)	0.329
**Total Knowledge Score (%)**	0.006 (-0.04 to 0.05)	0.797	0.13 (-0.24 to 0.50)	0.484
**Total Clinical Skills Score (%)**	0.02 (-0.02 to 0.96)	0.301	-0.11 (-0.39 to 0.16)	0.417
**Training at continuously monitored and supervised site**	-1.28 (-2.74 to 0.18)	0.086	17.77 (8.09 to 27.45)	<0.0001
**Presence of Key Enabling Indicators**	0.46 (0.12 to 0.80)	0.007	0.89 (-2.64 to 4.43)	0.618
**District:**				
**Mountains**	ref		ref	
**Hills**	1.37 (-0.26 to 2.99)	0.099	3.97 (-9.25 to 17.19)	0.553
**Terai**	2.90 (1.34 to 4.47)	<0.0001	11.57 (-0.09 to 23.22)	0.052
**Expected Deliveries per SBA**	0.05 (-0.01 to 0.11)	0.108	0.03 (-0.05 to 0.12)	0.417

## Discussion

Our study evaluated the competence and practice levels of a sample of skilled birth attendants in Nepal. We found that SBAs on average exhibit a deficiency of both knowledge and clinical skills, scoring poorly on assessments for both. In fact, across both knowledge and clinical skills, assessment scores failed to meet even the 80-percent standard that is required to pass training. This finding remained true no matter how recently training had concluded, suggesting not merely an attrition of skills but a failure to effectively learn them in the first place. This may indicate failure of the training program to effectively train the SBAs due to problems with the training methodology itself. We found also that SBAs are conducting very few deliveries, with only 7 percent meeting the minimal volume recommended to maintain competence by the WHO—and a substantial fraction performing an average of no monthly deliveries at all. Predictive of both their delivery volume and their knowledge and clinical skills scores was the quality of SBAs’ enabling environment: higher scores on the enabling environment index correlated with both higher knowledge and higher clinical skills assessment scores, in both hospitals and lower-level facilities. Higher enabling environment scores were also correlated with greater delivery volumes for SBAs in lower-level facilities. Finally, continuously monitored and supervised sites produced SBAs with higher average delivery volumes than other sites with lower levels of monitoring and supervision. Taken together, our findings suggest that current skilled birth attendant training alone might not suffice to ensure adequately productive and competent birth attendance in the field. Continuous monitoring and supervision of SBA training sites may improve the quality of training. Further support of health facilities via structural improvements that create a more enabling work environment may in turn improve the overall quality of work of skilled birth attendants in the field.

### Comparison with other studies

The first study to systematically assess SBA competencies was conducted from 2001 to 2002; a survey of 166 SBAs in four countries, it revealed similarly poor performance on clinical skills and knowledge. But it did not further analyze predictors of poor performance, the environmental factors under which SBAs work or their actual productivity in the field in terms of the number of deliveries conducted [5]. Other studies have built on this work, including one of 104 SBAs conducted just a year afterward in Nepal [6–10]. This study found that SBAs who underwent refresher trainings after some time in the field demonstrated greater clinical competence than those who did not, while also citing structural and environmental factors as barriers to SBA performance [6]. A more recent study in Nepal investigated this impact of environmental factors further, using semi-structured interviews and focus group discussions with SBAs to identify specific environmental constraints to maximal performance in the field [22]. None of these studies, however, explored predictive factors for the key outcomes of post-training delivery volume, knowledge, or clinical skills among SBAs. Nor has any study investigated SBA delivery volume as a core outcome measure, or used it as a surrogate for quality; yet we know from prior studies that obstetrical case load, and provider volume more generally, can be associated with better outcomes [19–21]. Critically, while at least a few have observed that SBAs are performing lower delivery numbers than expected, none has highlighted that a large proportion conduct a monthly average of zero deliveries, inspecting specifically the characteristics of this population. And while some have emphasized the importance of an adequate enabling environment for SBAs, none has sought to quantify this, nor rigorously correlated this with SBA competence and productivity. Our study, thus, contributes actionable insights about the efficacy of SBA training programs and the realities of SBAs’ capacity in the field.

### Conclusions and policy implications

Our study offers several important policy implications. Firstly, and perhaps most importantly, regular post-training follow-up is critical to assuring continued competence of SBAs. Indeed, none of our own findings would have come to light without the post-training follow-up conducted by Nepal’s National Health Training Center in coordination with NSI. In Nepal, the national policy since 2007 has been to subject at least 30 percent of SBA trainees to follow-up visits. Amidst resource constraints, however, only one such assessment prior to this study was conducted in 2009. If training programs are to be held accountable and SBAs are to be relied upon for quality maternal care, more frequent, nationally standardized post-training assessments must be imposed, in Nepal as in every country that invests in SBAs. Secondly, the high proportion of SBAs conducting zero average monthly deliveries challenges the value of quota-driven targets for SBA training: instead of struggling furiously to churn out as many new SBAs as possible, countries and their development partners should think instead about redesigning trainee selection, such that those who are already engaged in clinical care—like midwives and obstetric nurses—receive training, whereas those who are engaged in community work—like public health nurses—do not. Finally, the low numbers of deliveries conducted by formally trained SBAs begs the question: who is carrying out most of the deliveries in these settings? Should the focus of our training be on them? An estimated 23–43% of births in Nepal are attended by traditional birth attendants (TBAs). While the WHO has specifically excluded TBAs from their recommendation for skilled attendance at birth, they can play a valuable role where skilled care is available but underutilized, by serving as liaisons between rural women and health providers due to their existing social and cultural importance [23]. Training TBAs to provide prenatal and postpartum care, to recognize warning signs of pregnancy complications during delivery, and when to refer a woman to a health provider has the potential to improve maternal and newborn health outcomes in Nepal [24].

Of those SBAs in our sample who did attend births, the vast majority oversaw normal deliveries, with fewer than 15 percent managing complicated deliveries. This problem was particularly stark at the lower-level facilities, where three-quarters of SBAs who managed an average of zero complications were based. Possible reasons for this are manifold. As previous studies have reported, pregnant women might be choosing to bypass lower-level health facilities for hospitals, to seek more advanced obstetric care [25, 26]. Lower-level facilities might also offer fewer resources on average for managing complications, or staff less experienced SBAs. Both of these possibilities are supported by our findings: SBAs at lower-level facilities had accrued fewer months of nursing and SBA experience than their hospital counterparts. And lower-level facilities posted categorically poorer enabling environment scores than hospitals, reflecting at least partly a lower stock of basic emergency obstetric equipment that are integral to managing complicated deliveries. Interestingly, SBAs were split nearly evenly between permanent and temporary nurses—the latter of whom have acquired particular importance in rural Nepal, where the government has funded their recruitment to improve access to maternal health services [22]. Temporary nurses were found to be more productive than permanent ones; therefore, staffing these facilities with an even greater proportion of contract nurses might boost volume of both normal and complicated deliveries. Additionally, the vast majority of SBAs in our study were ANMs (91%), which in Nepal are maternal and child health workers, critical in rural areas, who have undergone a minimum of 18 months of training. The remaining 9% SBAs were staff nurses, who must undergo at least three years of training to receive their Proficiency Certificate Level in Nursing [27]. The lower level of training ANMs undergo compared to staff nurses and doctors could in part explain their poorer performance on the knowledge and clinical skills portions of the FEP.

To be effective, future SBA training policies should more carefully consider these inequalities, and design programs that more closely reflect the needs and constraints of different sites of work. Key to this effort will be equipping training programs with sufficient financial, technical and human resources to conduct monitoring and follow-up. Also important will be stocking health facilities with the equipment and basic amenities, like water and electricity that allow SBAs to fully exercise their skills: across our sample, SBAs ensconced in a positive enabling environment demonstrated greater mastery of clinical skills and a deeper fund of knowledge than those who were not. This leads us to conclude, if SBAs are supported from training through practice by a positive enabling environment, they are more likely to demonstrate sustained competence and productivity in the field.

### Limitations of this study

Though we assessed a sample of 511 SBAs in Nepal, we did not directly administer their training and we utilized secondary data which was not solely for the purpose of research. With an observational, descriptive study, we cannot ascribe causality. Moreover, we examined SBAs solely in Nepal; and though Nepal has historically served as a bellwether country for maternal health goals, it is possible that some of our results are idiosyncratic to its history and training program approach. As for our report of the enabling environment indicators, we are aware that the availability of a structural indicator, such as a partograph, does not necessarily translate into its proper use; therefore, high scores for some indicators might mask wide variations in clinical utility. Importantly, however, the enabling environment score is intended to be more a heuristic and foundational tool for understanding the resource limitations of facilities. From a policy perspective, accordingly, this should not alter the validity of our findings. Finally, maintaining internal and external validity is a challenge due to potential error or bias introduced in the sampling, measurement, data collection, and data analysis phases of the study.

## Conclusion

In spite of consistent and ongoing investments in the training of skilled birth attendants, in Nepal as around the world, our study suggests that these healthcare workers may not receive effective training, sufficient practice, or the appropriate experience to stay clinically competent and knowledgeable. The root of this problem seems to lie not merely in the attrition of skills over time, but also and fundamentally with the training programs themselves. Though many questions remain—for instance, under what circumstances a continuing education program might mitigate these outcomes—our study raises an important need to re-examine SBA training policies that advocate scale without evaluating quality. And it reminds us to relentlessly demand accountability, for both SBAs and the families they serve.

## Supporting information

S1 Table SBAs from different ecological zone, district, and health facility. (DOCX)

S2 Table Total deliveries per month in our sample. (DOCX)

S3 Table Total number of complications managed in our sample over a three month period. (DOCX)

S1 File SBA FEP tool. (PDF)

## References

[1] WHO, UNICEF, UNFPA, World Bank Group, United Nations Population Division. Trends in Maternal Mortality: 1990 to 2015.

[2] Alkema L, Chou D, Hogan D, Zhang S, Moller A-B, Gemmill A, et al Global, regional, and national levels and trends in maternal mortality between 1990 and 2015, with scenario-based projections to 2030: a systematic analysis by the UN Maternal Mortality Estimation Inter-Agency Group. The Lancet. 2015; 387: 462–74. 10.1016/S0140-6736(15)00838-7 PMC5515236 26584737

[3] United Nations Inter-Agency and Expert Group on MDG Indicators. The Millennium Development Goals Report 2015. United Nations, New York; 2015.

[4] Cha S. The impact of the worldwide Millennium Development Goals campaign on maternal and under-five child mortality reduction: “Where did the worldwide campaign work most effectively?” Global Health Action. 2017;10(1):1267961 10.1080/16549716.2017.1267961 28168932 PMC5328361

[5] Harvey SA, Blandón YC, McCaw-Binns A, Sandino I, Urbina L, Rodríguez C, et al Are skilled birth attendants really skilled? A measurement method, some disturbing results and a potential way forward. B World Health Organ. 2007;85(10):783–90. 10.2471/BLT.06.038455 PMC2636500 18038060

[6] Carlough M, McCall M. Skilled birth attendance: What does it mean and how can it be measured? A clinical skills assessment of maternal and child health workers in Nepal. International Journal of Gynecology & Obstetrics. 2005;89(2):200–208. 15847895 10.1016/j.ijgo.2004.12.044

[7] Bhuiyan AB, Mukherjee S, Acharya S, Haider SJ, Begum F. Evaluation of a Skilled Birth Attendant pilot training program in Bangladesh. International Journal of Gynecology & Obstetrics. 2005;90:56–60. 15936024 10.1016/j.ijgo.2005.03.031

[8] Partamin Kim YM, Mungia J, Faqir M, Ansari N, Evans C. Patterns in training, knowledge, and performance of skilled birth attendants providing emergency obstetric and newborn care in Afghanistan. Int J Gynaecol Obstet. 2012;119(2):125–129. 10.1016/j.ijgo.2012.05.030 22858205

[9] Ariff S, Soofi SB, Sadiq K, Feroze AB, Khan S, Jafarey SN, Bhutta ZA. Evaluation of health workforce competence in maternal and neonatal issues in public health sector of Pakistan: an Assessment of their training needs. BMC Health Services Research. 2010;10:319 10.1186/1472-6963-10-319 21110888 PMC3012669

[10] Ronsmans C, Endang A, Gunawan S, Zazri A, McDermott J, Koblinsky M, Marshall T. Evaluation of a comprehensive home-based midwifery programme in South Kalimantan, Indonesia. Tropical Medicine & International Health. 2001;6:799–810. 11679128 10.1046/j.1365-3156.2001.00780.x

[11] National Planning Commission, Government of Nepal. Nepal Millennium Development Goals Progress Report 2013. Government of Nepal, Kathmandu, Nepal; 2013.

[12] Central Bureau of Statistics. Nepal multiple indicator cluster survey 2014, final report. Central Bureau of Statistics and UNICEF Nepal, Kathmandu, Nepal; 2015.

[13] National Planning Commission, Government of Nepal. Sustainable Development Goals 2016–2030: National (Preliminary) Report. Government of Nepal, Kathmandu, Nepal; 2015.

[14] United Nations Population Fund. Post Training Follow-up for Skilled Birth Attendants: Review of Implementation Experiences. UNFPA; 2009.

[15] Goldstein R. A Monitoring and Evaluation Plan for the Core Competencies of Skilled Birth Attendants in Nepal. Capstone Collection. Paper 2492; 2012.

[16] Goyet S, Tamang L, Alvarez VB, Shrestha ID, Bajracharya K. Progress and challenges to introduce midwifery education in Nepal. The Lancet. 2017;389(10070):698–99. 10.1016/S0140-6736(17)30341-0 28229873

[17] Suvedi BK, Pradhan A, Barnett S, Puri M, Chitrakar SR, Poudel P, et al Nepal Maternal Mortality and Morbidity Study 2008/2009: Summary of Preliminary Findings. Kathmandu: Family Health division, Department of Health Services, Ministry of Health, Government of Nepal; 2009.

[18] WHO. The World Health Report: 2005: Make Every Mother and Child Count. Geneva: WHO Press 2005;79–102.

[19] Amato L, Colais P, Davoli M, Ferroni E, Fusco D, Minozzi S, Perucci CA. Volume and health outcomes: evidence from systematic reviews and from evaluation of Italian hospital data. Epidemiologia E Prevenzione. 2013;37(2–3):1–100. 23851286

[20] Kyser KL, Lu X, Santillan DA, Santillan MK, Hunter SK, Cahill AG, Cram P. The Association between Hospital Obstetrical Volume and Maternal Postpartum Complications. American Journal of Obstetrics and Gynecology. 2012; 207(1):42.e1–42.17. 22727347 10.1016/j.ajog.2012.05.010 PMC4362705

[21] Snowden JM, Cheng YW, Kontgis C, Caughey AB. The Association between Hospital Obstetric Volume and Perinatal Outcomes in California. American Journal of Obstetrics and Gynecology. 2012;207(6):478.e1–478.e7. 23174387 10.1016/j.ajog.2012.09.029 PMC3516613

[22] Morgan A, Jimenez Soto E, Bhandari G, Kermode M. Provider perspectives on the enabling environment required for skilled birth attendance: a qualitative study in western Nepal. Trop Med Int Health. 2014;19:1457–1465. 10.1111/tmi.12390 25252172

[23] Thatte N, Mullany LC, Khatry SK, Katz J, Tielsch JM, Darmstadt. Traditional birth attendants in rural Nepal: Knowledge, attitudes, and practices about maternal and newborn health. Glob Public Health. 2009;4(6):600–617. 10.1080/17441690802472406 19431006 PMC2762492

[24] Satishchandra DM, Naik VA, Wantamutte AS, Mallapur MD, Sangolli HN. Impact of training of traditional birth attendants on maternal health care: A community-based study. J Obstet Gynecol India. 2013;63(3):383–387. 24431684 10.1007/s13224-013-0457-4 PMC3889272

[25] Kruk ME, Hermosilla S, Larson E, Mbaruku GM. Bypassing primary care clinics for childbirth: a cross-sectional study in the Pwani region, United Republic of Tanzania. Bulletin of the WHO. 2014;92(4):246–253. 10.2471/BLT.13.126417 PMC3967574 24700992

[26] Salazar M, Vora K, Costa AD. Bypassing health facilities for childbirth: a multilevel study in three districts of Gujarat, India. Global Health Action. 2016;9: 10.3402/gha.v9.32178 27545454 PMC4992671

[27] Morrison J, Batura N, Thapa R, Basnyat R, Skordis-Worrall J. Validating a tool to measure auxiliary nurse midwife and nurse motivation in rural Nepal. Human Resources for Health. 2015; 13:30 10.1186/s12960-015-0021-7 25959298 PMC4429816

