# Supplementary material for: The quality of skilled birth attendants in Nepal: High aspirations and ground realities
Source: PLoS One. 2019 Apr 4;14(4):e0214577. doi: 10.1371/journal.pone.0214577 (PMC6448824; doi:10.1371/journal.pone.0214577)
Supplement: S1 Table — (DOCX) [file pone.0214577.s001.docx]

**S1 Table. SBAs from different ecological zone, district, and health facility.**

| Ecological Zone | Districts | Hospital | #SBA | PHC | #SBA2 | HP | #SBA3 | SHP | #SBA4 | Total # health institutions | Total # SBA |
| --- | --- | --- | --- | --- | --- | --- | --- | --- | --- | --- | --- |
| Mountain | Jumla | 1 | 3 | 1 | 1 | 8 | 14 | 0 | 0 | 10 | 18 |
|  | Taplejung | 1 | 4 | 1 | 1 | 8 | 10 | 3 | 3 | 13 | 18 |
|  | Bajhang | 1 | 8 | 1 | 5 | 14 | 20 | 0 | 0 | 16 | 33 |
| Hill | Dailekh | 1 | 3 | 3 | 5 | 13 | 18 | 0 | 1 | 17 | 27 |
|  | Pyuthan | 1 | 8 | 2 | 5 | 19 | 23 | 0 | 0 | 22 | 36 |
|  | Doti | 1 | 4 | 1 | 1 | 29 | 34 | 0 | 0 | 31 | 39 |
|  | Tanahu | 2 | 8 | 2 | 6 | 20 | 24 | 0 | 0 | 24 | 38 |
|  | Khotang | 1 | 8 | 0 | 0 | 5 | 6 | 4 | 4 | 10 | 18 |
| Terai | Udaypur | 2 | 8 | 1 | 2 | 7 | 16 | 3 | 2 | 13 | 28 |
|  | Dang | 2 | 23 | 3 | 7 | 17 | 22 | 0 | 0 | 22 | 52 |
|  | Parsa | 2 | 7 | 0 | 0 | 8 | 9 | 0 | 0 | 10 | 16 |
|  | Chitwan | 3 | 13 | 3 | 7 | 7 | 12 | 0 | 2 | 13 | 34 |
|  | Saptari | 2 | 13 | 2 | 12 | 19 | 27 | 0 | 2 | 23 | 54 |
|  | Banke | 1 | 10 | 3 | 7 | 23 | 35 | 0 | 0 | 27 | 52 |
|  | Bardiya | 1 | 8 | 3 | 8 | 16 | 27 | 5 | 5 | 25 | 48 |
| **Total** |  | **22** | **128** | **26** | **67** | **213** | **297** | **15** | **19** | **276** | **511** |
