# Supplementary material for: The quality of skilled birth attendants in Nepal: High aspirations and ground realities
Source: PLoS One. 2019 Apr 4;14(4):e0214577. doi: 10.1371/journal.pone.0214577 (PMC6448824; doi:10.1371/journal.pone.0214577)
Supplement: S2 Table — (DOCX) [file pone.0214577.s002.docx]

**S2 Table. Total deliveries per month in our sample.**

| **Ecological zone** | **Hospital** | **PHC** | **Health Post** | **Sub health post** | **Total** |
| --- | --- | --- | --- | --- | --- |
| Mountain | 63 | 36 | 121 | 0 | 324 |
| Hill | 199 | 67 | 363 | 9 | 945 |
| Terai | 1108 | 196 | 957 | 25 | **3459** |
