# Supplementary material for: The quality of skilled birth attendants in Nepal: High aspirations and ground realities
Source: PLoS One. 2019 Apr 4;14(4):e0214577. doi: 10.1371/journal.pone.0214577 (PMC6448824; doi:10.1371/journal.pone.0214577)
Supplement: S3 Table — (DOCX) [file pone.0214577.s003.docx]

**S3 Table. Total number of complications managed in our sample over a three month period.**

| **Ecological zone** | **Hospital** | **PHC** | **HP** | **SHP** | **Total** |
| --- | --- | --- | --- | --- | --- |
| **Mountain** | 4 | 1 | 3 | 0 | 8 |
| **Hill** | 25 | 5 | 20 | 2 | 52 |
| **Terai** | 445 | 49 | 262 | 5 | 761 |
| **Total** | 474 | 55 | 285 | 7 | **821** |
