# Supplementary material for: The quality of skilled birth attendants in Nepal: High aspirations and ground realities
Source: PLoS One. 2019 Apr 4;14(4):e0214577. doi: 10.1371/journal.pone.0214577 (PMC6448824; doi:10.1371/journal.pone.0214577)
Supplement: S1 File — (PDF) [file pone.0214577.s004.pdf]

Skill Birth Attendant Follow-up Enhancement Program

(SBA FEP)

SBA FEP TOOL
2016

Government of Nepal
Ministry of Health and Population
National Health Training Centre
Teku, Kathmandu Nepal

SBA Follow-Up and Enhancement Program
Assessment Tool

Background Information of Participant:

Name: ________________________________ Position: ______________________________

Type of contract: Permanent ( ) FHD ( ) VDC contract ( ) Other ( )

Age: ____________________ Phone No.: ____________________________

Education level: _____________ Council No.: __________________________

Total year of experience: ______________ Year & month of current workplace: ____________

SBA Training From : ____________________ Training completed year & month: __________

Name of current working District: _______________ Name of facility: __________________

Are you currently providing delivery service? Yes No

If not please mention date and department (remarks):_________________________________

Time to reach CEOC:_____________________ Total staff: _______________________

Number of Non SBA: ____________________ Number of SBA: ___________________

Number of Male Helper: _______________ Number of Female Helper: ________________

Grading of Facility (Base on coach individual perception)

Good _________ Sufficient _____________ Insufficient_____________

Date of assessment: __________________________

Name of coach: _____________________________ Signature of Coach: ____________________

PREFACE

Contents

Participant Interview

Knowledge Assessment

Knowledge Questionnaire

Clinical Assessment

Key Skills Checklists

- Conducting Normal Delivery
- Immediate Newborn Care
- Newborn Resuscitation

Clinical Decision Making

- Partograph &Decision making skill
- Management of Shock due to Postpartum Hemorrhage
- Referral

Optional (As per need)

- Vacuum Delivery
- Kangaroo Mother Care
- Post partum Hemorrhage- Condom Tamponade
- Management of Eclampsia

Infection Prevention Practice

- Preparation of Chlorine solution
- Putting on Sterile Glove
- Decontamination
- Cleaning Instrument
- Sterilization (Autoclave)

Enabling Environment

Checklist

- Labor Room Set-up
- Equipment
- Infrastructure
- Supplies
- Drugs
- IP Equipment and Supplies
- Health Care Waste Segregation & Dispose System
- Team Support
- Forms

Practical Experience

- Conducting normal deliveries
- Complicated procedures

FEP Evaluation

GUIDED INTERVIEW

Participant Interview

The purpose of the interview is to determine how the SBA training has impacted on the attitude, confidence and actual practice of the participant. Please ask probing questions by asking Why , How, What and get a few examples to find out the reasons for the responses.

1. How do you feel about conducting normal deliveries after SBA training? Why?

2. How do you feel about attending or managing complicated deliveries after SBA training? Why?

3. How often following major core skills are you using in your health facility? Please tick on the appropriate place.

Skills	Regularly	Sometimes	Never
a. ANC			
b. Family Planning			
c. Assisting Normal Birth (including AMSTL)			
d. Newborn Resuscitation			
e. Manual Removal of Placenta			
f. Vaginal and Perinal Tear			
g. Cervical Tear Repair			
h. Manual Vacuum Aspiration			
i. Vacuum Extraction			
j. Breech Delivery			
k. Shoulder Dystocia			
l. Management of Eclampsia			
m. Kangaroo Mother Care			
n. Condom Tamponade			
o. Referral Out			
p. Sterilization			
q. Waste Segregation			

4. What are the barriers or challenges to practice what you learned at SBA training?

   Please circle all the appropriate issues and explain the reason.

   a) Monitoring & Supportive supervision:

   b) Lack of HMC members support:

   c) Lack of community support:

   d) Others:

   e) No challenge:

5. How often do you get supportive supervision form district level?

6. Who come to your health facility for supervision?

7. Did you develop the Action Plan?

   Yes No (Please state the reason___________________)

8. If yes, what extent has this been implemented?

Implementation	Tick
Failure to implement the action plan	
Partially implement the action plan	
Completely implemented action plan	

9. If you implement the Action plan partially or completely, what are those?

a)

b)

c)

10. And if failure to implement, which challenges encountered to implement the Action Plan? Please list if it is not match with Q.N. 4.

KNOWLEDGE ASSESSMENT

1. KNOWLEDGE TEST

यो प्रश्नावलीहरु 27 core skills मा आधारित छन् । सबै SBA ले यो प्रश्नावलीहरु कसैसँग सल्लाह नगरी आफै गर्नुहोस् । तलका प्रश्नहरु राम्ररी पढ्नुहोस र मिल्ने उत्तरमा “क”, “ख”, “ग” वा “घ” मध्ये मा गोलो चिन्ह लगाउनुहोस ।

ANC:

१. मातृशिशु स्याहारको आधारभूत उद्देश्यमा के के पर्दछ ?

(क) आमा र नबजात शिशुको स्वास्थ परिणाम

(ख) जटिलता र समस्याको रोकथाम

(ग) जटिलता र समस्याको समयमा पहिचान तथा उपचार

(घ) माथिको सबै

२. मातृ तथा नवजात शिशु स्याहार का सामान्य सिद्धान्तहरु (सूत्रहरु):

(क) Clinical Decision Making, पारस्परिक संवन्ध कायम गर्ने सीप, संक्रमण रोकथाम, अभिलेख राख्ने र प्रेषण गर्ने

(ख) पारस्परिक संवन्ध कायम गर्ने सीप, संक्रमण रोकथाम, अभिलेख राख्ने र प्रेषण गर्ने

(ग) संक्रमण रोकथाम, अभिलेख राख्ने र प्रेषण गर्ने

(घ) माथिका कुनै पनि होईन

Patograph

३. Partograph मा के-के भरिन्छ?

क) FHS, Amniotic fluid , Moulding

ख) Cervical Dilation, Descend of the head, Contraction

ग) Vital Sign, Urine Output, Albumin in Urine

घ) माथिका सबै ।

४. यदि एउटी महिला प्रसुतिको Active Phase मा भर्ना भएमा Cervical Dilation हुँदा Partograph मा कहाँबाट शुरु गरिन्छ ?

क) Alert Line को देब्रेबाट ।

ख) Alert Line को दायाँबाट ।

ग) Alert Line बाट ।

घ) Action Line बाट ।

५.	प्रसवको असन्तोषजनक अवस्थाको पहिचान गर्न के के कुराले जनाउंछ ?

क) प्रारम्भिक चरण (Latent Phase) ८ घण्टाभन्दा लामो भएमा ।

ख) Partograph भर्दा पाठेघरको मुख खुलेको alert line को दायाँतिर action line तिर गएमा ।

ग) महिलाहरु जसले १२ घण्टा वा सो भन्दा लामो प्रसव पीडा भोगेपनि बच्चा जन्माउन नसकेमा ।

घ) माथिको सबै ।

६.	प्रसवको समयमा बच्चा निसासिए (Fetal distress) को जनाउने लक्षण तथा चिन्हहरु:

(क) Contraction नभएको बेला बच्चाको मुटुको घड्कन धेरै कम वा धेरै वढी हुनु

(ख) Amonnitic Fluid मा mecounium देखा पर्नु,

(ग) आमाको मुटुको धड्कन ठिक हुंदा पनि बच्चाको मुटुको धड्कन धेरै हुनु,

(घ) माथिका सवै

७.	बच्चा (Birth Asphyxia) हुन सक्ने अवस्थाहरु :

(क) दिन नपुगि बच्चा जन्मिनु,

(ख) बच्चा जन्मिनु अगाडि सालनाल निल्कनु,

(ग) प्रसवको समयमा Fetal distress हुनु,

(घ) माथिका सवै

८.	Augmentation गरिएको महिलालाई प्रसवको समयमा Fetal distress भएमा तुरुन्त के गर्नु पर्छ?

(क) यदि Oxytocin दिइएएको छ भने वन्द गर्नुपर्छ ।

(ख) यदि Oxytocin दिइएएको छ भने मात्रा वढाउनु पर्छ ।

(ग) यदि Oxytocin दिइएएको छ भने मात्रा घटाउनु पर्छ ।

(घ) माथिका कुनै पनि होईन ।

<u>Normal Delivery:</u>

९.	Third Stage of Labor को Active Management क्रमबद्ध तरिकाले कसरी गरिन्छ ?

क) Cord लाई बिस्तारै बाहिरतिर तान्ने, Fundal लाई massage गर्ने र 10 unit Oxytocin लगाउने

ख) नशाबाट Injection Oxytocin दिने Cord लाई बाँधेर काट्ने र Fundal लाई massage गर्ने

ग) Cord लाई बाँधेर काट्ने, नाल वा Cord लाई बिस्तारै बाहिरतिर तान्ने र सुई Ergometrive लगाईदिने र साल पाठेघर भित्र छुटेको छ वा छैन भनेर जाँच गर्ने ।

घ) Oxytocin सुई लगाईदिने, पाठेघरलाई एक हातले माथितिर धकेल्ने र अर्को हातले नाललाई विस्तारै तानेर Placenta लाई बाहिरतिर तान्ने। Placenta पुरै निस्केपछि तुरुन्तै आमाको पेट माथि हात राखेर विस्तारै पाठेघर भएको स्थानमा मालिस गर्ने ।

Vacuum Delivery:

१०. Vacuum Delivery गर्न सक्ने अवस्थाहरु:

क) पुरा महिना पुगेको (Full term fetus)

ख) पाठेघरको मुख पुरा खुलेको

ग) Fetal Head at least 0 station अथवा Syphilis Pubis 2/5 भन्दा तल

घ) माथिका सबै

११. योनीको परीक्षण गर्दा शिशुको तालुको पछिल्लो fontanelle लाई यस किसिमको महसुस गर्न सकिन्छ :

क) ठूलो र हिरा आकारको

ख) सानो र हिरा आकारको

ग) ठूलो र त्रिकोणात्मक आकारको

घ) सानो र त्रिकोणात्मक आकारको

Complicated procedure

१२. गर्भ अवस्थामा २८ हप्तापछि Vaginal Bleeding भएर आएको महिलालाई कसरी जाँच गरिन्छ?

(क) तुरुन्तै Vaginal examination गर्ने।

(ख) तुरुन्तै Vaginal examination नगर्ने ।

(ग) पेट छामेर मात्र जाँच गर्ने तथा आवश्यक परेमा Refer गर्ने ।

(घ) (ख) र)ग)

१३. Abruption Placenta भन्नाले के वुझ्नु हुन्छ ?

(क) बच्चा जन्मनु अगाडि सामान्य (Normal) अवस्थामा वसेको साल पाठेघरवाट छुट्टिनु ।

(ख) बच्चा जन्मिसकेपछि सामान्य (Normal) अवस्थामा वसेको साल पाठेघरवाट छुट्टिनु ।

(ग) पाठेघरको तल्लो भागमा साल वस्नु।

(घ) माथिका सवै

१४. बच्चा जन्मिएपछि पाठेघर राम्रोसँग खुम्चिए तापनि रक्तश्राव भइरहेको छ भने त्यसको कारण के हुन सक्दछ?

(क) पाठेघरको मुख च्यातिनु, र पाठेघर फुट्नु ।

(ख) पाठेघरको भित्री भागमा संक्रमण हुनु ।

(ग) योनीमा चोट लाग्नु वा च्यातिनु ।

(घ) क र ग ।

Newborn

१५. निसासिएको नवजात शिशुलाई bag and mask वाट resuscitation गर्दा:

क) सधै Oxygen प्रयोग गर्नुपर्छ

ख) Oxygen छ भने प्रयोग गर्ने

ग) Bag & Mask ventilation १ मिनेट मा ४० पटक

घ) Bag & Mask ventilation १ मिनेट मा ८० पटक

Eclampsia;

१६. गर्भअवस्थामा हुन सक्ने उच्च रक्तचाप संग सम्वन्धित:

क)अत्याधिक टाउको दुख्नु आखाँ धमिलो हुनु साथै Sever Epigastric Pain

ख) कम्पन तथा वेहोशी अवस्था

ग) पिसावमा Protein देखापर्नु

घ) माथिका सवै

१७. म्याग्नेसियम सल्फेट दिएपछि त्यसको Toxicity लाई अवलोकन गर्न हेर्नुपर्ने कुराहरु :

(क) नाडीको गति, श्वासप्रश्वास र रक्तचाप

(ख) श्वासप्रश्वासको गति, Patellar Reflex र पिसाबको मात्रा

(ग) शरीरको तापक्रम, नाडीको गति र श्वासप्रश्वास

(घ) BP हरेक चार चार घण्टामा लिने

१८. Magnesium Sulfate को Loading Dose के हो ?

(क) २०% म्याग्नेसियम सल्फेटको घोलको ४ ग्राम नशाबाट ५ मिनेटमा दिने, साथै ५०% म्याग्नेसियम सल्फेटको ५ ग्राम प्रत्येक फिलामा दिने ।

(ख) ५०% म्याग्नेसियम सल्फेटको घोलको ५ ग्राम १० मिनेटमा दिने ।

(ग) ५०% म्याग्नेसियम सल्फेटको घोलको २ ग्राम प्रत्येक फिलाको मासुमा दिने ।

(घ) माथिको कुनै पनि होइन ।

१९. Post Partum Depression का चिन्ह तथा लक्षणहरु के के हुन ?

(क) निन्द्रा नलाग्नु,

(ख) धेरै तथा अनावश्यक रुपमा दुखी देखिनु र आफ्नो वच्चाको स्याहार नगर्ने

(ग) आत्माबल कमजोर हुने तथा चिन्तित हुनु,

(घ) माथिका सवै

Infection Prevention:

२०. हात धुनको लागि पानी नभएको खण्डमा:

क) अलिकति स्पिरिट लगाएर पुछ्ने।

ख) ६०%-९०% को १००ml रेक्टीफाइट स्पिरिटमा, २ ml ग्लसिरिन मिसाउने र ३.५ ml लिएर हात धुने।

ग) हात नधोएर पनि काम गर्न सकिन्छ।

घ) माथिका कुनै पनि होइन।

२१. दुषण निवारण गर्नाको उद्देश्यहरु के के हुन ?

(क) प्रयोग गरिसकेको औजार, उपकरणहरुमा रेहका एचआई. हेटाटाहिटिज र अन्य जिवाणुहरुलाई नष्ट गर्नु

(ख) विरामीलाई संक्रमण हुनबाट जोगाउनु,

(ग) सामान सफा गर्ने व्यक्तिलाई सुरक्षित वनाउंछ ।

(घ) माथिका सवै

२२. स्वास्थ्य संस्थामा प्रयोगमा आएका औजारहरुलाई तुरुन्तै दुषण रहित कसरी गर्न सकिन्छ ?

(क) साबुन पानीले धोएर २ घण्टासम्म उमाल्ने

(ख) 0.5% Chlorine मा १० मिनेटसम्म डुबाउने

(ग) सर्फ पानीमा ३० मिनेट डुवाउने।

(घ) सर्वप्रथम साबुन पानीले राम्ररी धोएर 0.5% Chlorine मा १० मिनेट डुबाउने

२३. High Level Disinfection (HLD) कसरी गरिन्छ ?

क) पानीमा उमालेर मात्र

ख) उम्लेको पानीमा २० मिनेट उमाल्ने वा २० मिनेट सम्म वाष्पिकरण गर्ने वा रसायनिक घोलमा २० मिनेटसम्म डुबाउने

ग) दुषणरहित घोलमा २० मिनेटसम्म डुबाउने

घ) कुनैपनि होइन्

२४. नेपाल सरकारको Health Care Waste Management Guideline 2014 अनुसार स्वास्थ्य संस्थाको हानिकारक फोहरहरुको व्यवस्थापन गर्न...................गर्नु पर्दछ।

क) फोहरको प्रकार अनुसार छुट्टा छुट्टै संकलन गर्ने व्यवस्था

ख) सवै प्रकारको फोहर संकलन गर्ने भाँडोहरुमा फोहरको प्रकारको चिन्ह वुझ्ने गरि लेवल लगाई सवै विभाग वा वार्डहरुमा अनिवार्य राख्ने व्यवस्था

ग) सवै प्रकारको फोहर संकलन गर्ने भाँडोहरुमा फोहरको प्रकारको चिन्ह वुझ्ने गरि लेवल लगाई सवै कर्मचारी लाई जानकारी गराउने व्यवस्था

घ) माथिका सवै

२५. दक्ष प्रसुतीकर्मी (SBA) भन्नाले मान्यता प्राप्त स्वास्थ्यकर्मी जस्तैः डाक्टर वा नर्स, मिडवाईफ जो निम्न लिखित शिपहरुमा निपुण गराउनको लागि तालिम प्राप्त हुन्छ............

क) सामान्य गर्भावस्था, प्रसुती, प्रसुती पश्त्यातका अवस्थाहरुको व्यवस्थापन गर्ने, मातृ तथा नवशिशुमा देखा परेका जटिलताहरुको पहिचान गर्ने र प्रेशण गर्ने

ख) सामान्य गर्भावस्था, प्रसुती, प्रसुती पश्त्यातका अवस्थाहरुको व्यवस्थापन गर्ने, मातृ तथा नवशिशुमा देखा परेका जटिलताहरुको पहिचान गर्ने, व्यवस्थापन गर्ने र प्रेशण गर्ने

ग) सामान्य गर्भावस्था, प्रसुती, प्रसुती पश्त्यातका अवस्थाहरुको व्यवस्थापन गर्ने, मातृ तथा नवशिशुमा देखा परेका जटिलताहरुको पहिचान गर्ने, र प्रेशण गर्ने

घ) सामान्य गर्भावस्था, प्रसुती, प्रसुती पश्त्यातका अवस्थाहरुको व्यवस्थापन गर्ने

<u>Supervision:</u>

२६. सुपरीवेक्षण प्रकियामा निम्न लिखित कुराहरु समावेश गरिन्छः

क) स्वास्थ्य संस्थाको निरिक्षण गर्ने र Job Description अनुसारको व्यत्तिगत कार्यसम्पादनको सुपरिवेक्षण गर्ने

ख) गल्तीहरु पत्ता लगाउने र त्यसमा सहभागी हुनेहरुलाई दण्ड दिने

ग) स्टाफहरुको कार्यसम्पादनमा सुधार ल्याउनको लागि स्टाफहरुलाई पथ प्रदशन गर्ने, तालिम दिने, सहयोग गर्ने र उत्साह प्रदान गर्ने

घ) क र ग

<u>Comments from coach:</u>

Name | Gravida | Para | Hospital number

Date of admission | Time of admission | Ruptured membranes | hours

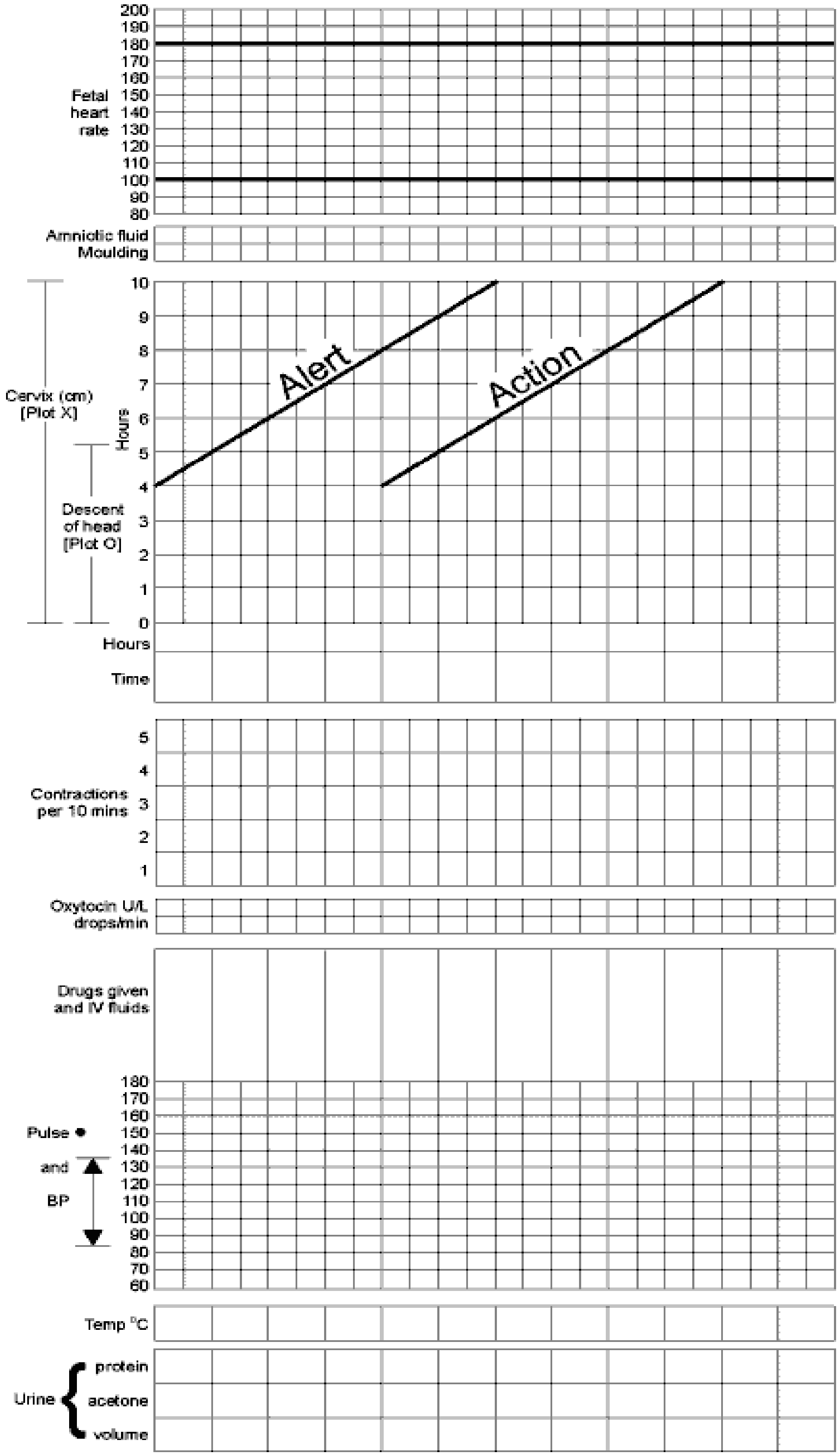

PARTOGRAPH

Instruction:
Provide a not plotted partograph to the participant and request to plot the information as information given by coach. The below statement should be provided to the participants by coach with asked question as given below.

१. Initial plotting exercise

२. Fetus को अवस्था कस्तो छ थाहा पाउँनको लागि पार्टोग्राफ अनुसार के के हेरिन्छ र भरिन्छ ?

३. व्यथाको अवस्था थाहा पाउँनको लागि पार्टोग्राफ अनुसार के के हेरिन्छ र भरिन्छ?

४. आमाको अवस्था थाहा पाउँनको लागि के के जाचँ गरिन्छ र सोधेर पार्टोग्राफमा भरिन्छ?

५. FHS /contraction कति कति घण्टामा हेनुपर्छ? ....

P/V exam कति कति घण्टामा गरिन्छ?...................

वच्चाको टाउको तल झरेको (Descend of the head) कति कति घण्टामा हेरिन्छ र भरिन्छ ....

६. Plotting exercise

७. तपाईले भर्नु भएको Partograph हेर्नुहोस र यदि तपाई non CEOC site मा हुनुहुन्छ भने तपाईले भर्नु भएको ९ वजे सम्म्को partograph plotting मा देखेअनुसार तपाई के गर्नु हुन्छ?

Applied for CEONC site

८. त्यस्तै यदि तपाई CEOC site मा हुनुहुन्छ भने सो Partograph मा देखेअनुसार तपाईले के गर्नु हुन्छ?

९. Augmentation भनेको के हो र यो कहाँ गर्न सकिन्छ? यो कसरी गरिन्छ साथै Augmentation गरेको महिलालाई PV गर्नु परेमा कुन कुन वेला गर्न सकिन्छ?

१०. माथिको Partograph ले देखाए अनुसार (९ वजे सम्मको) सो महिलालाई Augmentation गर्न सकिन्छ कि सकिदैन?

११. Plotting exercise

१२. तपाईले Plot गरेको Partograph हेर्नुहोस (१२:३० वजे सम्मको) र यसमा तपाईले के के समस्या देख्नु भयो?

१३. यस्तो अवस्थामा अव तपाईले के के गर्नु पर्दछ?

Comments from coach:

Is the SBA competent in filling Partograph?

- Yes, the SBA is competent
- No, the SBA is not competent

MAKING 0.5% CHLORINE SOLUTION

STEP/TASK	Before coaching	After coaching
1. Get all the necessary equipment ready red plastic tub (bata), bucket of water, chlorine powder packet (Virex), apron, utility gloves, eye shield, cap, 1lt. jug.		
2. Get 10-15 liters of water for adequate preparation		
3. Put on cap,plastic apron, face mask and protective eye shields if available		
4. Put on utility gloves and fold the gloves cuffs towards the palm		
5. Put 1 liter of water in a bucket in which you are mixing the Virex powder.		
6. Read instructions on the chlorine powder packet.		
7. Cut the tip of the Virex packet with scissors. (hole should be big enough to pour out the Virex)		
8. Pour Virex powder into the bucket gently so it does not blow into the air and reach your eyes.		
9. Gently mix the powder in the water with the help of the gloved hands until the chlorine powder completely disappears.		
10. Add water gently into the bucket to a total of 9 liters		
11. Pour the 0.5% chlorine solution into the container that will be used in the clinic		
12. Keep the chlorine container in a proper place.		
13. Clean gloved hands in clean water before removing.		
14. Put gloves in a proper place to dry.		
15. Ask/ observe the dusting LR bed after each delivery.		

Source: IP and Health care waste management course notebook for trainers, NHTC 2015

<u>Comments from coach:</u>

Is the SBA competent in making chlorine solution?

- o Yes, the SBA is competent
- o No, the SBA is not competent

PUTTING ON STERILE GLOVES

STEP/TASK	Before coaching	After coaching
1. Open outside packet of gloves and remove inner packet. Carefully open and expose gloves without touching the inside of the packet.		
2. Wash hands well with soap and water. Dry with clean towel or air dry.		
3. Pick up one glove touching only the wrist inside portion.		
4. Insert hand into the glove as far as possible without touching the outside of the glove.		
5. Take the partially gloved hand and insert the fingers between the palm and wrist, touching only the outside (sterile) portion of the gloves.		
6. Insert the other hand into the glove as much as possible.		
7. Use both hands to put hands into the gloves fully being careful not to touch the inside of the gloves or skin.		

Source: IP and Health care waste management course notebook for trainers, NHTC 2015

<u>Comments from coach:</u>

Is the SBA competent in Sterile Gloving?

- Yes, the SBA is competent
- No, the SBA is not competent

CONDUCTING NORMAL DELIVERY

STEP/TASK<br>(Some of the following steps/tasks should be performed simultaneously)	Before coaching	After coaching
**Getting Ready/Assisting Birth**		
1. Prepare the necessary equipment.		
2. Encourage the woman what is going to be done, listen to her and respond attentively to her questions and concerns.		
3. Tell the woman what is going to be done, listen to her and respond attentively to her questions and concerns.		
4. Provide continual emotional support and reassurance, as feasible.		
5. Put on personal protective barrier.		
**Assisting Birth**		
6. Wash hands thoroughly with soap and water and dry with a clean, dry cloth or air dry.		
7. Put high- level disinfected or sterile surgical gloves on both hands.		
8. Clean the women's perineum with antiseptic solution wiping from front to back.		
9. Place one steril e drape from delivery pack under the women's buttocks, one over her abdomen and use one drape to receive the baby.		
**Birth of the Head**		
10. Ask the women to paint or give only small pushes with contractions as the baby's head is born.		
11. As the pressure of the head thins out of the perineum, control the birth of the head with the fingers of one hand, applying a firm, gentle downward(but not restrictive) pressure to maintain flexion, allow natural stretching of the perineal tissue and prevent tears.		
12. Use the other hand to support the perineum using the gauze to compress and allow the hand to crown slowly and be born spontaneously.		
13. Wipe the mucous (and membrane) from the baby's mouth and nose with a clean gauze.		

14. Feel around the baby's neck to ensure the umbilical cord is not around the neck.<br>• If the cord is around the neck but is loose, slip it over the baby's head.<br>• If the cord is tight around the neck, clamp the cord with the two artery forceps, placed 3cm apart and cut the cord between the two clamps.		
**Completing the Birth**		
15. Allow the baby's head to turn spontaneously.		
16. After the head is turns, place a hand on each side of the baby's head upward toward the mother's abdomen as the posterior shoulder is born over the perineum.		
17. Lift the baby's head anteriorly to deliver the posterior shoulder.		
18. Move the topmost hand from the head to support the rest of the baby's body as it slides out.		
19. Place the baby on the mother's abdomen (if the mother is unable to hold the baby , ask her birth companion or an assistant to care for the baby).		
20. Thoroughly dry the baby and cover with clean, dry cloth and remove wet cloth.		
21. Assess breathing while drying the baby and if it does not breath immediately, begin resuscitative measures.		
22. Ensure the baby is kept warm and skin – skin contact on the mother's chest and cover the baby (ies) and proceed with active management of the third stage.		
**Active management of Third Stage of Labor**		
23. Give oxytocin 10 units I. M		
24. Delayed cord clamping		
25. Clamp and cut the umbilical cord:<br>• Tie the cord at about 3cm and 5cm from the umbilicus.<br>• Cut the cord between the ties.		
26. clamp the cord close to the perineum and hold the clamped cord and the end of the clamp in one hand.		
27. Place the other hand just above the pubic bone and gently apply counter traction (push upwards on the uterus) to stabilize the uterus and prevent uterine inversion.		

28. Keep tight tension on cord and wait for a strong uterine contraction (2 to 3 min).		
29. When the uterus become rounded or the cord lengthens, very gently pull downward on the cord to deliver the placenta.		
30. Continue to apply counter traction with other hand.		
31. If the placenta doesnot descend during 30 to 40 seconds of controlled cord traction, relax the tension and repeat with the next contraction.		
32. As the placenta delivers, hold it with both hands and twist slowly so the membranes are expelled intact:<br>• If the membranes do not slip out spontaneously, gently twist them into a rope and move up and down to assist separation without tearing them.		
33. Slowly pull to complete delivery.		
**Massage Uterus**		
34. Immediately massage uterus through woman's abdomen. Show the woman how to massage her uterus to maintain contraction.		
35. Repeat uterine massage every 15 min minutes for first 2 hours.		
36. Ensure that uterus does not become relaxed.		
37. Immediate breast feeding.		
**Examination of placenta**		
38. Hold placenta in palm of hands, with maternal side facing upwards: check whether all lobules are present and fit together.		
39. Now hold the cord with one hand and allow placenta and membrane to hang down:<br>• Insert fingers of other hand inside membranes, with fingers spread out and inspect membranes for completeness.<br>• Note position of cord insertion.		
40. Inspect cut end of cord for presence of two arteries and one vein.		
**Examination of Vagina and perineum for Tears**		
41. Gently separate the labia and inspect lower vagina for laceration/ tears.		
42. Gently cleanse the perineum with warm water and a clean cloth.		

43. Apply the clean pad or cloth to the vulva.		
44. Remove all wet/ soiled bed linen and dispose of appropriately.		
45. Ensure the woman is comfortable and cover her with blanket.		
**Immediate Post-Partum Care**		
46. Measure the woman's BP, pulse every minutes till 2 hours		
47. Continue uterine massage every 15 minutes till 2 hours.		
48. Help initiate early breast feeding:<br>• Encourage first feeding with in first hour of birth by leaving baby in skin-skin-to skin contact with mother.<br>• Give assistance at first feed, if required, to ensure baby is correctly position and attached to breast.<br>• Allow unrestricted time at breast once the baby starts to suckle.		
49. Review woman's complication readiness plan and update to reflect postpartum/newborn needs:<br>• Advise woman and her family to entact plan if any danger signs show.		
50. Provide health messages and counseling about:<br>• Keep baby dry and covered with clean, warm cloth.<br>• Maintain skin-to-skin contact.<br>• Do not bath baby for first 24 hours.<br>• If the room is cold, add blanket/covering to mother and baby.		
51. Continue uterine massage (see above).		
**Continuation of Immediate Newborn Care**		
52. Help initiate early breastfeeding (see above).		
53. Securely attach an identification label to baby's wrist or ankle.		
54. Provide eye care with boiled and cooled water if necessary.		
55. Apply chlorohexidine ointment on baby's cord from cut end of cord to the base with gloved hand.		
56. Prepare for newborn physical examination.		

Post -Procedure Task		
57. Place any contaminated items(e.g, swabs) in a plastic bag or leak-proof covered waste container.		
58. Decontaminate instruments by placing in a container filled with 0.5% chlorine solution for 10 minutes.		
59. Needles and syringes:<br>Place in a puncture – resistant sharps container.		
60. Immerse both gloved hands briefly in a container filled with 0.5% chlorine solution then remove gloves by turning them inside out:<br>• If disposing of gloves (examination gloves and surgical gloves that will not be reused), place in a plastic bag or leak-proof covered waste container.<br>• If reusing surgical gloves, submerge in 0.5% chlorine solution for 20 minutes for decontamination.		
61. Wash hands thoroughly with soap and water and dry with clean, dry cloth or air dry.		
62. Recording and Reporting.		

Comments from coach

1. Is the SBA competent in normal delivery?
    - o Yes, the SBA is competent
    - o No, the SBA is not competent

NEW BORN RESUSCITATION

**STEP/TASK**<br>**(Some of the following steps/tasks should be performed simultaneously)**	**Before coaching**	**After coaching**
**<u>Knowledge Questions</u>**<br>1. Prepare equipment and supplies for resuscitation before EVERY birth:<br>• bag and mask,<br>• suction apparatus with tubbing & catheter,<br>• DeLee Suction<br>• soft clothes.		
2. At every birth ask or look for the following to determine the need for resuscitation:<br>• Meconium not present?<br>• Breathing or crying?<br>• Good muscle tone?<br>• Term gestation?<br>If the answer to each question above is "YES", do routine immediate newborn care steps.<br>**Note: If the baby needs more support, do "Initial Steps of Resuscitation".**		
**<u>Pre-Procedure</u>**<br>3. Inform mother and other family member about resuscitation if baby is not breathing or aspexia.		
4. Quickly clamp and cut the cord. Quickly wrap the baby in the clean, dry, warm cloth and keep covered except for the face and chest.		
5. Move the baby on its back on a clean, warm surface (resuscitation corner)		
6. Quickly wrap the baby in the clean, dry, warm cloth and keep covered except for the face and chest.		
<u>During Procedure</u><br>7. Position of the baby:<br>Put the wrapped baby on its back with the rolled cloth under the shoulders to slightly extend the head. To extend the head put the clothes two finger thick under the baby's shoulders.		
8. Remove the suction tube from packet. If there is no suction machine use Dr. Lee machine.		
9. Clear airway (as necessary) by suctioning the mouth first and then the nose. Introduce 5 cm catheter into the baby's mouth and suction while withdrawing catheter.		

10. Introduce catheter into each nostril 3 cm and suction while withdrawing catheter. Be especially thorough with suctioning if there is blood or meconium in the baby's mouth and/or nose.		
11. Reposition the baby. Place the mask on the baby's face so that it covers the mouth and nose to form a seal<br>• Size 1 mask for normal weight newborn and<br>• Size 0 for a small newborn		
12. Ventilate the baby 2 times.		
13. Use enough pressure to squeeze the bag so that you see a gentle rise and fall of the chest.<br>If the baby's chest is rising, ventilate the baby 40 times in 1 minute.		
14. If the baby's chest is not rising:<br>• Ensure the correct position<br>• Ensure air is not leaking from mask<br>• Squeeze full bag for ventilation<br>• Re-suction		
15. Evaluate after 1 minute of resuscitation and inform mother and family about possible results.<br>• If the baby is not breathing, continue to ventilate 40 times in 1 minute then re-evaluate.<br>• If baby start breathing stop resuscitation.<br>• If baby still not breathing continue resuscitation.		
16. **After 20 minutes**<br>• Stop resuscitation if baby does not breath and inform mother and family about results.<br>• If the baby is gasping continue for another 10 minute.<br>**After 30 minutes**<br>• Stop resuscitation if baby does not breath baby might be dead<br>• If the baby is gasping stop resuscitation and inform mother and family that the baby could not be saved.<br>**Wait till half an hour of activity to declare death of baby.**		

<u>Comments from coach</u>

Is the SBA competent in New Born Resuscitation?

- ○ Yes, the SBA is competent
- ○ No, the SBA is not competent

DECONTAMINATION

STEP/TASK<br>(Some of the following steps/tasks should be performed simultaneously)	Before coaching	After coaching
1. Leave on surgical or examination gloves post-procedure or put on utility gloves.		
2. Place all instruments in 0.5% chlorine solution for 10 minutes for decontamination immediately after completing the procedure		
3. Dispose of waste material in leak proof container or plastic bag		
4. Decontamination examination or OR table or other surface contaminated during the procedure by wiping them with 0.5% chlorine solution		
5. Remove instruments from 0.5% chlorine solution after 10 minutes and place them ijn water		
6. Clean instruments immediately (GO TO CLEANING) or continue to soak in water until cleaning can be done.		
7. If wearing surgical or examination gloves, immerse both gloved hands in 0.5% chlorine solution. Remove gloves by turning inside out.		
8. If disposing of gloves, place in lick proof container or plastic bag.<br>If reusing gloves, submerge in 0.5% chlorine solution for 10 minutes for decontamination. If wearing utility gloves, do not remove until instrument cleaning is finished.		

Source: IP and Health care waste management course notebook for trainers, NHTC 2015

<u>Comments from coach:</u>

Is the SBA competent in Decontamination?

- Yes, the SBA is competent
- No, the SBA is not competent

CLEANING(INSTRUMENTS)

**STEP/TASK**<br>**(Some of the following steps/tasks should be performed simultaneously)**	**Before coaching**	**After coaching**
1. Wearing utility gloves on both hands, place instruments in a basin with clean water and mild, non-abrasive detergent.		
2. Completely disassemble instruments and /or opens jaws of jointed items.		
3. Wash all instrument surfaces with a brush or cloth until visibly clean (hold instruments under water while cleaning)		
4. Thoroughly clean serrated edges (e.g., jaws of hemostat) of instruments using small brush.		
5. Wash surgical gloves in soapy water, cleaning inside and out.		
6. Rinse all surfaces with clean water until no shop or detergent remains.		
7. Towel dry instruments using clean, dry towel or allow them to air dry.		
8. Hang surgical gloves up and allow to air dry. when first side is dry, reverse and re-hang to dry completely.		
9. After cleaning all items, remove utility gloves and allow them to air dry.		

Source: IP and Health care waste management course notebook for trainers, NHTC 2015

Comments from coach:

Is the SBA competent in Cleaning (Instruments)?

- Yes, the SBA is competent
- No, the SBA is not competent

STERILIZATION (AUTOCLAVING)

**STEP/TASK**<br>**(Some of the following steps/tasks should be performed simultaneously)**	**Before coaching**	**After coaching**
1. Double wrap instruments in freshly laundered cloth or paper using envelope or square wrap technique.		
2. Fold up cuffs of surgical gloves, place gauze or paper inside glove and under fold of cuff and wrap in cloth or paper.		
3. Place wrapped gloves thumbs up in wire basket on their sides.		
4. Arrange instruments packs on an autoclave cart or shelf. Place in autoclave chamber to allow free circulation and penetration of steam to all surfaces.		
5. Put autoclave tape (Indicator tape in packed instrument)		
6. Sterilize wrapped items for 30 minutes. Time with clock at121EC(250EF) and 106kpa(15bls/in2).		
7. Wait until pressure gauge reads zero before opening lid or door14-16cm(5-6 inches). This may take 20-30 minutes.		
8. Allow packs to dry completely before removal.This may take up 30 minutes.		
9. Place sterilized packs on a surface padded with paper or fabric to prevent condensation.		
10. Allow packs to reach room temperature before storing		
11. Record sterilization conditions(time, temperature, and pressure) in log book.		

Comments from coach:

Is the SBA competent in Sterilization (Autoclaving)?

- o Yes, the SBA is competent
- o No, the SBA is not competent

MANAGEMENT OF SHOCK DUE TO PPH (HYPOVOLEMINC SHOCK)

Instruction:

The below statement (Italic fond) should be provided to the participants by trainer with asked question given in the following Table (Right side of the column) And Expected response from participants are given in the middle column. And Expected response from participants is given in the middle column. Based on the responses, please mark the key response as “C” or “N” or “NA” in the given left side column**.**

**Statement to be explained by trainer to participant:**<br>*Mrs. L is a 36-year-old multigravida who has five children. Her husband, who tells you that she gave birth at home with the help of a tradition birth attendant, has carried her into the hospital. The birth attendant told him that the placenta delivered easily and completely immediately after birth, but Mrs. L has been bleeding “too much” since then. The family tried numerous things to help Mrs. L before bringing her to the hospital, but she continues to bleed “too much”.*		
**Question to be asked**	**Expected Response**	**Score**
What do you do?	1. Shouts for help to urgently mobilize all available personnel	
	2. Evaluates Mrs. L immediately for shock, including vital signs (pulse, blood pressure, and respiration rate), level of consciousness, color and skin temperature	
	3. Tells Mrs. L (and her husband) what is going to be done, listen to her and responds attentively to her questions and concerns.	
	4. Turns Mrs. L on her side, if unconscious or semi conscious, and keeps the airway open.	
**Statement to be explained by trainer to participant:**<br><br>B. On examination you find that Mrs. L’s pulse is 120 beats/minute, blood pressure 84/50 mm Hg and respiration rate 34 breaths/minute. Her skin is cold and clammy.		
**Question to be asked**	**Expected Response**	**Score**
What is Mrs L ‘s Problem?	5. States that Mrs. L is in shock.	
After seeing problem what will you immediately do?	6. Start an IV infusion, using a large-bore cannula and normal saline or Ringer’s lactate at a rate of 1 L in 15-20 minutes	
	7. While starting the IV, collects blood for appropriate tests (hemoglobin, and bedside clotting test for coagulopathy)	
	8. Looks for the cause of shock (hypvolemic or septic) by palpating the uterus for firmness and tenderness, assessing the amount of blood loss	

**Statement to be explained by trainer to participant:**<br>C. you find that Mrs. L's uterus is soft and not contracted. Her clothing from the waist down is blood soaked.		
**Question to be asked**	**Expected Response**	**Score**
	9. Ensure whether woman get Oxytocin 10 unit I/M	
What will you do?	10. Starts a second IV infusion and gives 20 units oxytocin in 1 L of fluid at 60 drops/minute (10 units/bottle)	
	11. Massages Mrs. L's uterus to stimulate a contraction	
	12. Starts oxygen at 6-8 L minute if available	
	13. Catheterizes bladder	
	14. Covers Mrs. L to keep her warm	
	15. Elevates legs	
**Question to be asked**	**Expected Response**	**Score**
What are Mrs L's main problems?	**16.** States that Mrs. L reportedly lost "too much" blood after childbirth and considerable blood loss is evident on her clothes.	
	17. Mrs. L's uterus is soft and not contracted	
	18. Determines that Mrs. L's is in shock due to postpartum hemorrhage with atonic uterus.	
**Question to be asked**	**Expected Response**	**Score**
How do you know when a woman is in shock?	19. Pulse greater than 110 beats/minute, systolic blood pressure less that 90 mm Hg; cold clammy skin; pallor; respiration rate greater than 30 breaths per minute; anxious and confused or unconscious	
What should be monitor and how often need to be monitor the woman having shock due to atonic uterus?	20. Monitor pulse, blood pressure and respiration in every 15 minutes	
	21. Continues to check that uterus remains contracted in every 15 minutes	
	22. Check vaginal bleeding (continuous or stop)	
**Statement to be explained by trainer to participant**<br><br>D. After 15 minutes the uterus is firm and bleeding has stopped, but Mrs. L's pulse is still 116 beats/minute, blood pressure 88/60 mmHg and respiration rate 32 breaths/minute.		
**Question need to ask**	**Expected response**	**Score**
What will you do now?	**23.** Gives another liter of fluid to ensure 2 L are infused within an hour of starting treatment	
	24. Continues to give oxygen at 6-8 L/minute	
	25. Monitor pulse and blood pressure in every 15 minutes	
	26. Continues to check that uterus remains contracted in Every 15 minutes	

Statement to be explained by trainer to participant:		
After another 15 minutes, the uterus is still firm and there is no further bleeding. Mrs. L's pulse is 90 beats/minute, blood pressure 100/60 mm Hg and respiration rate 24 breaths/minute.		
**Question need to ask**	**Expected response**	**Score**
What will be your action?	27. Adjusts rate of IV infusion to 1L in 6 hrs	
	28. Continue to check to ensure that uterus remains contracted	
	29. Continue to monitor pulse and blood pressure	
	30. Check that urine output is 30ml/hour or more	
**Statement to be explained by trainer to participant:** *Mrs. L's condition has stabilized. Twenty-four hours later, her hemoglobin is 9 g/dL*		
What will you do now?	31. Begins ferrous sulphata 120mg and folic acid 400mcz by mouth daily for three months.	

Comments from coach:

Is the SBA competent in Eclampsia?

- Yes, the SBA is competent
- No, the SBA is not competent?

Referral Procedure

**STEP/TASK**<br>**(Some of the following steps/tasks should be performed simultaneously)**	**Before coaching**	**After coaching**
1. Explains to the family about the condition and the reason for referred to another center to the woman as appropriate.		
2. Ask the need for transportation management		
**Preparation Document and necessary action**		
3. Documentation on Patient condition at the time of arrival		
4. Finding of the examination		
5. Treatment given		
6. Check vital sign		
7. Present condition (Stabilize the condition)		
8. Write the name and contact number of service provider on the document prepare for refer.		
9. Suggest to accompanied the visitors who are able/capable for emergency situation such as blood donation.		
10. Informed to the referred site for alert & prepare for readiness		
11. Assist woman to reach the ambulance if necessary service provide need to be accompany up to refer site and hand over.		
12. Follow- up patient's condition: Telephone follow up		

Note: Go to Annex 1

<u>Comments from coach:</u>

Is the SBA competent in Referral?

- ○ Yes, the SBA is competent
- ○ No, the SBA is not competent

Optional

VACUUM DELIVERY

STEP/TASK<br>(Some of the following steps/tasks should be performed simultaneously)	Before coaching	After coaching
**<u>Getting Ready</u>**		
1. Prepare the necessary equipment		
2. Tell the woman what is going to be done, listen to her and respond attentively to her questions and concerns		
3. Provide continual emotional support and reassurance, as feasible		
4. Review to ensure that the following conditions for vacuum extraction are present:<br>a. Term Fetus<br>b. Vertex presentation<br>c. Head at least at 0 station or no more than 2/5 palpable above the symphysis pubis<br>d. Cervix fully dilated		
5. Make sure an assistant is available		
6. Put on personal protective barriers		
7. Procedure task		
8. Wash hands thoroughly with soap and water and dry with a clean, dry cloth or air dry		
9. Put high level disinfectant or steril surgical gloves on both hands		
10. Clean the vulva with antiseptic solution		
11. Catheterize the bladder, if necessary		
12. Check all concerns on the vacuum extractor and test the vacuum on a gloved hand		
Skill Questions<br>13. Assess the position of the fetal head by feeling the sagittal suture line and fontanelles.		
14. Identify the posterior fontanelle.		
15. Perform episiotomy if necessary for proper placement of the cup.<br>• If episiotomy is not necessary for placement of cup, delay until the head stretches the perineum or the perineum interferes with the axis of		

traction.		
16. Apply the largest cup that will fit, with the center of the cup over the flexion point, 3 cm anterior to the posterior fontanelle.		
17. Check the application and ensures that there is no maternal soft tissue (cervix or vagina) within the rim of the cup.<br>• If necessary, release pressure and reapply cup.		
18. Have the assistant create a vacuum of 0.2kg/cm^2 (147mmhg) negative pressure with the pump and check the application of the cup.		
19. Increase the vacuum to 0.8kg/cm^2 (500-588mmhg) negative pressure and check the application of the cup.		
20. After the maximum negative pressure has been applied start traction in the line of the pelvic axis and perpendicular to the cup:<br>• If the fetal head is tilted to one side or not flexed well, traction should be directed in a line that will try to correct the tilt or deflexion of the head (i.e. to one side or the other, not necessarily in the midline).		
21. With each contraction, apply traction in a line perpendicular to the plane of the cup rim:<br>• Place a gloved finger on the scalp next to the cup during traction to assess potential slippage and descent of the vertex.		
22. Between each contraction have assistant check:<br>• Fetal heart rate<br>• Application of the cup.		
23. Failure of vacuum extraction if:<br>• Fetal head doesn't advance with each pull.<br>• Fetus is under delivered after 3 pull with no decent<br>• Cup slips of the head twice at the proper direction of pull with a maximum negative pressure.		
24. When the head has been delivered, release the vacuum, remove the cup and complete the delivery.		
25. Check the birth cannel for tears following delivery and repair if necessary.		
26. Provide immediate postpartum and newborn care, as require.		

Comments from coach:

Is the SBA competent in Vacuum delivery?

- Yes, the SBA is competent
- No, the SBA is not competent

KANGAROO MOTHER CARE (KMC)

STEP/TASK<br>(Some of the following steps/tasks should be performed simultaneously)	Before coaching	After coaching
**Getting Ready**		
1. Prepare the necessary equipment.		
2. Greets the mother/ guardian and make him/her questions.		
3. Explains what she is going to do.		
4. Encourages the mothers/ guardian to ask Question and address his/her questions.		
5. Washes hands with soap and water and air dries it.		
6. Dress the baby in cap, socks and nappy/diapers.		
7. Explains that the person who will be doing KMC should wear loose dress that has opening in front.		
8. Make traditional wrap ready (unfolds 3 meters long wrap and hold it by dividing half in the middle.)		
9. Opens the front part of blouse or upper half part of the dress.		
10. Positioning the baby in KMC position<br>• Place baby on mothers/ guardian chest between her breasts in an upright position.<br>• The head should be turned to one side and in a slightly extended position. The slightly extended head position keeps the airway open and allows eye to eye contact between the mother/ guardian and her baby.<br>• The back and buttocks of the baby is supported by one hand during this time.<br>• Baby's hands are placed above the mother's/ guardian's chest.<br>• Baby's feet are placed below the mother's / guardians breast i.e. frog like position.		
11. Puts the center of the wrap over the baby on the mother's / guardians chest.		
12. Wraps both ends of the cloth around the mother/ guardian under his/ her arms to her back and tie knot securely.		

*NB: Baby should not slip out when the mother stands up or moves around.		
13. Supports the baby's head by pulling the wrap just up to the ear of the baby.		
14. Cover mother/ guardian and baby with a shawl.		
15. Documentation of Findings.		

Comments from coach:

Is the SBA competent in Kangaroo Mother Care?

- o Yes, the SBA is competent
- o No, the SBA is not competent

CONDOM TAMPONADE

STEP/TASK<br>(Some of the following steps/tasks should be performed simultaneously)	Before coaching	After coaching
**Getting Ready**		
1. Prepare the necessary equipment.		
2. Tell the woman (and her support person) what is going to be done, listen to her, and respond attentively to her questions and concerns.		
3. Provide continual emotional support and reassurance, as feasible		
4. Ensure the bladder is empty, catheterize it if necessary .		
5. Give prophylativ antibiotics		
6. Put on personal protective barriers.		
**Insertion**		
7. Wash hand and forearm thoroughly and put on High – level disinfected or sterile surgical gloves(use elbow-length gloves, if available).		
8. Place condom over the foley catheter leaving a small portion of the condom beyond the tip of catheter.		
9. Using a a sterile suture or a string, tie the lower end of condom on the foley catheter. Tie should be tight enough to prevent leakage of saline solution but should not strangulate catheter and prevent inflow of Water.		
10. Place a Sims speculum in the posterior Vaginal wall. Hold the anterior lip of cervix with the sponge or ring forceps. Using an antiseptic technique place the condom end high into the uterine cavity by digital manipulation or with the aids of forceps.		
11. Connect outlet of foley catheter to an IV set connected to a saline bag or bottle of saline. Inflate condom with saline to about 300-500ml(or to amount at which no further bleeding is observed.)		
12. Fold over the end of Catherter and tie with a threat or a cord clamp when desired volume is achieved and bleeding is controlled.		
13. Maintain in- situ for 12-14 hours if bleeding controlled and client is stable.		

14. Continue uterotonic infusion: 20 IU oxytocin in 1000 ml saline solution, 60 drops/ minute		
15. Continue to monitor client closely, resuscitate and / or treat shock necessary.		
16. If bleeding is not controlled with in 15 minutes of initial insertion of condom tamponade abandon the procedure and seek surgical intervention immediately.		
**Deflation**		
17. When no further bleeding has occurred and the client has been stable for at least 12 to 24 Hours slowly deflate condom by letting out 50-100 ml of saline every hour.		
18. Re- inflate to previous level if bleeding reoccurs while deflating.		
19. Cord the catheter while deflating.		
**Post-Procedure Tasks**		
**20.** Remove gloves and discard the mini leak-proof container or plastic bag.		
21. Wash hands thoroughly		
22. Regularly monitor vaginal bleeding, take the women's vital signs and make sure that the uterus firmly contracted.		
23. Recording and Reporting.		

<u>Comments from coach:</u>

Is the SBA competent in Condom Tamponade?

- Yes, the SBA is competent
- No, the SBA is not competent

MANAGEMENT OF ECLEMPSIA

Instruction:

The below statement (Italic fond) should be provided to the participants by trainer with asked question given in the following Table(Right side of the column) And Expected response from participants are given in the middle column. Based on the responses, please mark the key response as “C” or “N” or “NA” in the given left side column**.**

Mrs C is 23 years old. She is 37 weeks pregnant. Her mother has brought her to the health center because she developed a sever headache and blurred vision this morning.

Question to basked	SN	Expected Response	Responses
What will you do?	1	Greet the pregnant woman.	
	2	Take full history of pregnant woman	
	3	Check the vital sign and abdominal examination	

Mrs C blood pressure is 160/110mmhg. She has protein uria 3+. FHS is normal. History of sever headache, blurred vision.

Question to be asked	SN	Expected Response	Responses
What will be your action?	4	Administer loading dose of Magnesium Sulphate	
	5	Explain about the condition to patient visitors with stating the need for referral to the CEONC site.	
	6	Prepare for referral. (see referral procedure)	

<u>Statement to be explained by trainer to participant:</u>

A. Mrs. G is 16 years old and is 37 weeks pregnant. This is her first pregnancy. She has presented to the labor unit with contractions and says that she has had a bad headache all day. She also cannot see properly. While she is getting up from the examination table, she falls back onto the pillow and begins to have a convulsion.

Question to be asked	SN	Expected Response	Responses
What will you do?	7	Shout for help to mobilize all available personnel	
	8	Check airway to ensure that it is open, and turns Mrs. G onto her left side	
	9	Protect her from injury, but does not attempt to restrain her.	
	10	Has one of the staff member start oxygen at 4-6 L/min.	
	11	Has one of the staff members help to take Mrs G’s vital sign (BP, Pulse, Respiration)	
	12	Check her level of consciousness	
	13	Open I/V Line	
	14	Prepare & gives magnesium sulphate 20% solution, 4g IV over 5 minutes.	

	15	Follows promptly with 10g of 50% magnesium sulphate solution, 5g in each buttock deep IM injection with 1mL of 2% lignocaine in the same syringe.	
	16	Check Vital sign and necessary action for stabilizing the condition	
In such situation what you call for this condition?	17	Eclampsia with convulsion	
If you are working in Non-CEOC site; what will you do?	18	Explain to the family what is happening and talk to the woman as appropriate	
	19	Give Nephidine 10 mg orally to keep diastolic BP between 90-100mmHg	
	20	Prepare for referral	
	21	One has insert an indwelling catheter	
If you are working in CEOC site what will you do?	22	Same as above plus	
	23	Maintain a strict fluid balance chart	
	24	Continuously monitor for BP, plus, respiration, patellar reflexes and fetal heart	
	25	Monitor for the development of Pulmonary Edema by assaulting lung's bases for rales	
	26	Assess Mrs G's cervix to determine whether it is favorable or unfavorable	
	27	Monitor for mother and fetal condition and plot in partograph and review frequently	
	28	Continue maintenance dose of MGSo4 -5gm with 1 ml of 2% lignocaine in alternate buttocks every 4 hours for 24 hours after the last convulsion or delivery whichever occur last	
	29	Monitor for Magnesium toxicity before giving the next maintenance dose.<br>• Respiration more than 16/min<br>• Urinary output more than 30 ml/hour<br>• Patellar reflex present	
	30	State the childbirth should occur within 12 hours of the onset of Mrs G's convulsions	

<u>Comments from coach:</u>

Is the SBA competent in Eclampsia Management?

- Yes, the SBA is competent
- No, the SBA is not competent

ENABLING ENVIRONMENT

Facility Checklist:

For each of the following points, please put number and mark a "√" for the answer in the appropriate column.

Equipment	Available		If yes, is it working condition	
	Yes	No	Yes	No
**1. Labor Room Set-up**<br>• Placement of Delivery bed				
• Newborn Corner Set- up				
• Readiness for resuscitation				
**2. Delivery set**				
**3. Standard Delivery sets (Minimum 3 sets)**<br>• Sponge holding forcep-1				
• Cord clamp forceps – 2				
• Cord cutting scissor – 1				
• Wrapper – 4				
• Galley pot (small bowl)				
• Bowl – 1				
Note: Standard Delivery sets				
**4. Tear Repair set:**<br>• Episiotomy Scissors				
• Tooth Forcep				
• Needle Holder				
• Suture				
**5. Newborn resuscitation set:**<br>• Neonatal Ambu bag(No 0 or 1)				
• Suction: DeLee or foot/Suction tubbing				
**6. Vacuum set with silicon cup in different size**				

**7. Cervical tear repair set:**<br>• Sponge holder – 4				
• Sim's spaculum – 2				
• Needle holder – 1				
• Tooth / Non-tooth forceps - 1 each				
• Scissors-1				
**8. MVA set**<br>• Spacullum (Double Valve)-1				
• Sponge Holding Forcep -1				
• Galipot -1				
• Kidney Tray				
• Volsellum				
• MVA Syringe				
• Cannula in different size				
**9 PPIUCD Set**				
Tray-1				
Ring Forceps/Sponge Holder				
Placenta Kelly's forceps				
Sims Speculum				
Galley Pot				
**10. IUCD Set**<br>• Tray for instrument				
• Sponge holder forcep				
• Duck bill speculum				
• Valsellum				
• Galley-pot				
• IUCD				
• Scissors				
• Uterine Sound				
**General Equipment**				
11. Gudal Airway				

12. Fetoscope				
13. Baby weighing machine				
14. Stethoscope				
15. Blood pressure Instrument				
16. Thermometer				
17. Oxygen				
18. Suction				

**INFRASTRUCTURE**	**Yes**	**No**	**If no, please write reason**
19. Room for ANC/FP			
20. Room for delivery			
21. Electricity			
22. Electric back-up system (generator or inverter)			
23. Running water (Water supply within the premises)			
24. Toilet in Labor Room			
25. Toilet outside the Labor Room			
26. Road access			
**Supplies**	**Yes**	**No**	**If no, please write reason**
27. IV cannula & IV set			
28. Catgut # 1			
29. Plain catheter			
30. Refrigerator			
31. Foley's Catheter			
32. Surgical Gloves			
33. Gauze, cotton			
34. Wall clock			
35. Linen (wrapper for delivery set & baby)			
36. Soap and detergent			

DRUG - not expired	Yes	No	If no, please write the reason
37. Oxytocics			
**38.** Inj. Ampicillin			
**39.** Metronidiazol			
40. Gentamycin			
41. Paracetamol			
42. Local Anesthetic (1%, 2%)			
43. Mesoprostal			
44. Magnesium sulfate (50%)			
45. 10 ml Syringe			
46. 20 ml Syringe			
47. Inj. Ergometrine			
48. Cap Depin			
49. Antidote of Magnesium sulfate(Calcium Gluconate)			
50. Vitamin A			
51. Antiseptic solution – Betadine			
**Infection Prevention Equipment / supplies**	**Yes**	**No**	**If no, please write the reason**
52. 15 ltr plastic bucket or bowl for chlorine solution			
53. Chlorine			
54. Utility Gloves			
55. Eye Protection (Eye glass)			
56. Close shoes			
57. Apron			
58. Container for sterile gauze, cotton pieces (drum)			
59. Sterilization equipment (autoclave)			
60. Sterilization equipment (steamer)			

Health Care Waste Segregation & Dispose System

Dispose of sharp instrument	Yes	No	If no, please write the reason
61. Needles, scalpel blades and other sharp objects are disposed of in a puncture-proof container immediately after use.			
62. Discard placenta in Red bucket with cover			
**63. Waste segregation**			
a) Black- Non risk waste (Rotten)			
b) Red- Blood and blood contaminated			
c) Blue- Non risk waste (Plastic)			
d) Dark Blue- Bottle			
e) Green- Paper only			
64. Color bucket in Labor Room			
65. Color bucket in Emergency Room (ER)/ Out Patient Department (OPD)			
**Process of waste disposal**			
66. Protective barriers for transportation (Apron, utility gloves, mask and cap)			
67. Transportation- use of 4 wheel trolley			
68. Time of disposal- Early morning/ Late Evening (as per necessary)			
69. Land field- Properly designed and managed			
70. Incinerator properly functioning- When<br>• When it is use?<br>• Who use the incinerator?			
71. Placenta pit- Utilization			
72. wash hands thoroughly with shop and water and dry with clean, dry cloth or air dry.			

Source: IP and Health care waste management course notebook for trainers, NHTC 2015

Team Support	Yes	No	Comment
73. Is there HDC/ HFOMC?			
74. Is there regular (Quarterly) meeting of HDC/ HFOMC?			
75. Are in-charge organize staff meeting prior to HDC/HFOMC meeting ?			
76. Is MNH or other issues taken into action?			
77. If yes, what are they? ____________________			
**Forms**			
78. Partograph			
79. Check the filled partograph (at least 3)			
80. EOC Referral Slip (# of Referral /month)			
81. Maternity Register Book /MNH Register			

PRACTICAL EXPERIENCE

DELIVERIES		Procedure performed	Ref. In	Ref. Out
1	Conducting normal deliveries			
2	Breech deliveries			
3	Vacuum delivery			
4	Twins delivery			
5	Shoulder Dystocia			
6	Assist in Cesarean section			
**COMPLICATED PROCEDURE**				
7	Episiotomy & tear repair			
8	Management of PPH			
9	Neonatal resuscitation			
10	Management of Pre/eclampsia			
11	Cervical tear repair			
12	Blood transfusion			
13	Management of MRP			
14	MVA			
15	PPIUCD /IUCD Insertion			
16	Management of APH			
17	Management of ectopic pregnancy			
18	Post Abortion Care (PAC)			
19	Others			
**Base on Health Facility**				
20	Total delivery of pervious year			
21	Total delivery by SBAs (Last 3 months)			
22	Total delivery by Non SBA (Last 3 months)			
23	Case referral out			
24	Case referral in			
25	Total LSCS in last year			
26	Total LSCS in last 3 months			

EVALUATION OF SBA FEP PROGRAM

For SBA Participant

Please give the answer the question for the future improvement of SBA FEP

1. What is the most useful area of this follow-up program?

2. What is the least useful area of this follow-up program?

3. Please give your opinion how do you like the tool & the process to be improved?

KNOWLEDGE ASSESSMENT

1. Knowledge Test

This questionnaire is based upon the 27 core skills. The SBA should complete the questionnaire independently without consulting literature or colleagues.

Read the following questions and mark the best answer with a circle around the letter “a”, “b”, “c” or “d”.

ANC

1. The goals of basic maternal and newborn care include:
   a) Healthy outcomes for the mother and newborn
   b) Prevention of complications and problems
   c) Early detection and treatment of complications and problems
   d) All of the above.

2. The basic component of maternal and newborn care is**:**

   a. Clinical decision making, interpersonal communication skills, infection prevention, recording, reporting & referral
   b. interpersonal communication skills, infection prevention, recording, reporting & referral
   c. Infection prevention, recording, reporting & referral
   d. None of above

Partograph

3. What parameters need to be fill in Partograph?
   a. FHS, Amniotic fluid , Moulding
   b. Cervical Dilation, Descend of the head, Contraction
   c. Vital Sign, Urine Output, Albumin in Urine
   d. All of above

4. If a woman is admitted during the active phase of labor, cervical dilation is initially plotted on the Partograph:
   a) To the left of the alert line
   b) To the right of the alert line
   c) On the alert line
   d) On the action line

5. Unsatisfactory progress of labor should be suspected if:
   a) The latent phase is longer than 8 hours
   b) Cervical dilatation is plotted to the right of the alert line on the partograph
   c) The woman has been experiencing labor pain for 12 hour or more without giving birth
   d) All of the above

6. Fetal distress during labor is indicated by:
    a) A very slow or rapid fetal heart rate in the absence of contraction
    b) Meconium staining in amniotic fluid in case of vertex presentation
    c) A rapid feral heart rate in the absence of rapid maternal heart rate
    d) All of the above.

7. Conditions, which may lead to birth asphyxia, include:
    a) Pre-term birth
    b) Prolapsed cord
    c) Feral distress during labor.
    d) All of the above

8. If fetal distress occur under the augmented woman, what you do immediately?
    a) Oxytocin should be stopped if it is being administered
    b) The rate of oxytocin should be increased if it is being administered
    c) The rate of oxytocin should be decreased if it is being administered
    d) None of the above

<u>Normal Delivery</u>

9. What are the appropriate order of steps in active management of the third stage of labor:
    a) Controlled cord traction, fundal massage and 10 units of oxytocin
    b) Intravenous oxytocin, cord clamping and cutting, and fundal massage
    c) Cord clamping and cutting, controlled cord traction, ergometrine administration, and inspection to be sure the placenta intact.
    d) Intramuscular injection of oxytocin, controlled cord traction with counter traction to the uterus, and uterine massage.

<u>Vacuum Delivery:</u>

10. Condition for vacuum delivery is/ are:
    a) Full term fetus
    b) A fully dilated cervix
    c) Fetal head at least at 0 station or not more than 2/5 above the symphysis pubis.
    d) All of the above
11. On vaginal examination, the posterior fontanelle will feel:
    a) Large and diamond shaped
    b) Small and diamond shaped
    c) Large and triangular in shape
    d) Small and triangular in shape

Complicated procedure

12. How the assessment of a woman who presents with vaginal bleeding after 28 weeks of pregnancy should do?
    a) Immediate vaginal examination
    b) Not to do immediately Vaginal examination
    c) Simple abdominal examination and if necessary refer
    d) (b) and (c)

13. Abruption placenta is:
    a) The detachment of normally located placenta from the uterus **before** the fetus is delivered
    b) The detachment of normally located placenta from the uterus **after** the fetus is delivered
    c) Localization of placenta in the lower uterine segment covering the cervical OS
    d) All of above

14. Veginal bleeding immediately after birth in the presence of a well contracted uterus; what may the reasons?
    a) Cervical tear & uterine rupture
    b) Endometritis
    c) Perinal trauma or tear
    d) A and C

Newborn

15. While doing resuscitation to the asphyxiated baby using bag and mask:
    a) Always use Oxygen
    b) Use oxygen if available
    c) Bag and Mask ventilation 40 times per minute
    d) Bag and Mask ventilation 80 times per minute

Eclampsia

16. Associated sign and symptom during pregnancy induce hypertension:
    a) Sever Headache, Blurred Vision and Sever Epigastric Pain
    b) Convulsion and unconscious
    c) Protein in urine
    d) All of above

17. The woman should be closely monitored for the sign of toxicity of magnesium sulfate:
    a) Pulse, respiration, blood pressure
    b) Respiration rate, patellar reflection and urinary output
    c) Temperature, pulse and respiration
    d) Glasgow coma scale

18. What is loading dose of magnesium sulfate?
    a) 4gm of 20% magnesium sulfate solution IV over 5 minutes followed by 5 gm of 50% magnesium sulfate IM in each buttock
    b) 5 gm of 50% magnesium sulfate solution IV over 10 minutes
    c) 2 gm of 50% magnesium sulfate IM over both buttocks
    d) None of above

19. What are the sign and symptoms of postpartum depression?
    a) Unable to sleep
    b) Excessive or inappropriate sadness or guilt and neglect own baby
    c) Feelings of worthlessness or anxiousness
    d) All of the above

<u>Infection Prevention</u>

20. Wash hand while water is not available……
    a) Scrub hands with spirit
    b) Scrub hands with 3-5 ml solution containing the mixture of 2ml Glycerin in 100 ml of 60%-90% Rectified Spirit
    c) No need for hand washing
    d) None of above

21. What is the aim of decontamination?
    a) Decrease the chances of transmission of HIV, Hepatitis B C from contaminated instruments
    b) Protect patients from infection transmission
    c) Protect worker from infection transmission
    d) All of above

22. How could the contaminated instrument would be decontaminated immediately in the health facility?
    a) Washed with soap and water and boiled for hours
    b) Soaked in0.5% chlorine solution for 10 minutes
    c) Soaked in0.5% chlorine solution for 30 minutes
    d) Washed with soap and water and soaked in 0.5% chlorine solution for 10 minutes

23. High-level disinfection (HLD) can be achieved by:
    a) Boiling in water only
    b) Boiling in water or soaking in disinfectant solution for 20 minutes.
    c) Soaking in disinfectant solution for 20 minutes only
    d) None of the above

24. According to Nepal Government Health Care Waste Management Guideline 2014 the harmful waste from health facility should be manage by….
    a) Separate collection of waste based on types of waste
    b) All containers should be labeled based on type of waste and keep in all departments and wards compulsory
    c) All containers should be labeled based on type of waste and should be inform to all the staff
    d) All of above

25. A skilled birth attendant (SBA) is an accredited health professional, such as a midwife, doctor or nurse, who has been educated and trained to proficiency in the skills needed to:
    a) Manage normal pregnancies, childbirth, the postnatal period and identify and refer complications in women and newborn
    b) Manage normal pregnancies, childbirth, the postnatal period and identify, manage and refer complications in women and newborn
    c) Manage normal pregnancies, childbirth, the postnatal period and refer complications in women and newborn
    d) Manage normal pregnancies, childbirth, the postnatal period

<u>Supportive Supervision</u>

26. Supervision is a process that involves:
    a) Inspecting facilities and supervision of individual performance based on Job Description
    b) Finding fault or errors and then punished those involve
    c) Guiding, helping, coaching and encouraging staff to improve their performance.
    d) (a) and (c)

Annex 1

REFERRAL
(Note for coach: Please tear this page and give to participant)

**A. Antenatal Cases- Obstetric Case**		**A. Antenatal Cases- Non-Obstetric Case**	
**SN**	**Condition**	**SN**	**Condition**
1	Hyperemesis	1	Pregnancy with known case of heart disease , hypertension and?
2	Pre-eclampsia	2	Diabetes
3	Previous CS	3	Thyriod disorders
4	APH	4	SLE and other auto immune disorders
5	Placenta previa	5	Severe anemia in late pregnancy
6	Multiple pregnancies	6	Jaundice complicating pregnancy
7	Malpresentation/abnormal lie	7	Fever
8	Post- term pregnancy	8	Seizure disorders with pregnancy
9	IUGR,PROM	9	Psychiatric illness
10	IUD	10	COPD and other respiratory problems
**11**	**PROM**	11	HBsag/ HIV AIDS(if management not available)

**C. Emergency Referral Of Mother (Depends upon Sites)**			
**SN**	**Condition**	**First Trimester**	
		**SN**	**Condition**
1	Premature labour (refer to where neonatal intensive care is avaible)	1	Heavy bleeding
2	Premature rupture of membrane	2	Acute abdomen

3	PPH and third stage complication after first aid measures like IV crystalloids, condom tamponade, continuous bladder drainage and oxytocin drip.	3	Suspected ruptured ectopic
4	Eclampsia after giving loading dose of Magnesium sulphate with proper documentation., Severe pre-eclampsia, Gestational hypertension	4	Sever infection
5	Antepartum Hemorrhage		
6	Ruptured ectopic	**Second Trimester**	
7	Cord prolapse	1	Heavy bleeding
8	Failed induction in case of CEONC	2	Severe hyperemesis
9	Prolong labour/ Obs labour	**Third Trimester**	
10	Incomplete abortion (from BC)	1	Failed induction
11	Trauma, High vaginal tear, cervical tear, uterus rupture	2	Prolonged pregnancy (>41 weeks) has been added
12	Mal presentation and position	**Post-partum**	
13	Fetal distress	1	Retained placenta (if CCT fails at BC)
14	Any indication for CS	2	Uncontrolled PPH
15	Any abnormality in pregnancy	3	Puperial pyrexia, eclampsia, sudden collapse, suspected DVT.

D. Indication for referral of Newborn baby

SN	Condition	SN	Condition
1	Preterm <34 weeks	10	Pathological abdominal distension/bilious vomiting
2	LBW<1.8Kg ??	11	Sick newborn, poor feeding/poor activity
3	Birth asphyxia	12	Birth trauma (brachial plexus palsy, fractures)
4	Jaundice appearing within 24 hours;	13	Central cyanosis

5	Congenital malformation (trachea oesophagealfistula, gastro-intestinal atresia, diaphragmatic hernia, ruptured meningo-myelocoele, ectopiavesicate)	14	Respiratory distress or respiratory rate> 60 per Minute with cyanosis /grunt/severe chest retractions/in drawing.
6	Any bleeding manifestation in spite of vitamin k administration.	15	Failure or pass meconium in 24 hrs
7	Bulging anterior fontanelle	16	Failure or pass meconium in 24 hrs
8	Blood in stools	17	Convulsions
9	Apnoea	18	Failure or pass urine in 48 hrs
